# Foehn–cold pool interactions in the Inn Valley during PIANO IOP2

**DOI:** 10.1002/qj.3735

**Published:** 2020-01-29

**Authors:** M. Haid, A. Gohm, L. Umek, H. C. Ward, T. Muschinski, L. Lehner, M. W. Rotach

**Affiliations:** ^1^ Department of Atmospheric and Cryospheric Sciences University of Innsbruck Innsbruck Austria

**Keywords:** cold‐air pool, foehn, heat budget, multiple Doppler wind lidars, shear flow instabilties, turbulent erosion

## Abstract

A case‐study is presented of a south foehn emanating from the Wipp Valley, Austria, which encountered a cold‐air pool (CAP) in the Inn Valley near the city of Innsbruck. The analysis is based on data collected during the second Intensive Observation Period of the Penetration and Interruption of Alpine Foehn (PIANO) field experiment. Foehn was initiated on 3 November 2017 by an eastward moving trough and terminated in the afternoon of 5 November 2017 by a cold front passage. On two occasions, reversed foehn flow deflected at the mountain ridge north of Innsbruck penetrated to the bottom of the Inn Valley. The first breakthrough occurred in the afternoon of 4 November 2017. It was transient and locally limited to the northwest of the city. The second (final) breakthrough occurred in the morning of 5 November 2017 and was recorded by all surface stations in the vicinity of Innsbruck. It started with a foehn air intrusion to the northeast of Innsbruck and continued with the westward propagation of the foehn–CAP boundary along the valley. Subsequently observed northerly winds above the city were caused by an atmospheric rotor. A few hours later and prior to the cold front passage, the CAP pushed back and lifted the foehn air from the ground. During both nights, shear flow instabilities formed at the foehn–CAP interface, which resulted in turbulent heating of the CAP and cooling of the foehn. However, this turbulent heating/cooling was partly compensated by other mechanisms. Especially in the presence of strong spatial CAP heterogeneity during the second night, heating in the CAP was most likely overcompensated by negative horizontal temperature advection.

## Graphical abstract

A case‐study is presented of a south foehn emanating from the Wipp Valley, Austria, which encountered a cold‐air pool in the Inn Valley near the city of Innsbruck. The goal is to investigate small‐scale processes that govern the breakthrough of foehn in the valley based on observations collected during the PIANO field experiment. The study evaluates the contribution of shear flow instabilities (illustrated in the figure) and temperature advection to the heat budget of the cold‐air pool.

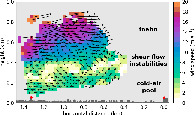

## 1 INTRODUCTION

Foehn in Alpine valleys is characterized by a complex, transient wind field and strong turbulence. This has implications for air pollution in valleys (e.g., Seibert *et al*., 2000; Furger *et al*., 2005; Gohm *et al*., 2009) and poses a hazard to aviation (e.g., Gohm *et al*., 2008; Chan and Hon, 2016). For example, in the Inn Valley aircraft have to approach Innsbruck Airport from opposite directions prior to and after foehn breakthrough to ensure safety (e.g., Angerer *et al*., 2018). Therefore an accurate forecast of foehn breakthrough and interruption in the valleys is crucial for aviation safety and air quality prediction. However, in order to improve the forecast, processes which control these transient phases of foehn onset and decay have to be better understood. Often prior to foehn breakthrough, the atmosphere in Alpine valleys is characterized by a stably stratified cold‐air pool (CAP) at the valley bottom and potentially warmer foehn flow aloft. The CAP forms during night‐time, when the boundary layer is cooled due to long‐wave outgoing radiation (Whiteman, 2000). Before foehn is able to penetrate into the valley, the CAP needs to be removed. Gubser and Richner (2001) mention three potential mechanisms of CAP erosion during foehn: (1) bottom‐up heating of the CAP, (2) top‐down heating by turbulent mixing, and (3) CAP displacement. However, these processes are not exclusive to foehn in the Alps. They also play a role in cold‐pool studies in other regions in the absence of an upper‐level foehn flow (e.g., Price *et al*., 2011; Lareau *et al*., 2013).

*Process 1*. Surface sensible heat flux associated with solar radiation heats the valley atmosphere and weakens its stability. Hoinkes (1949) hypothesized that foehn breakthrough is only possible when the potential temperature in the valley matches the foehn temperature. For the Owens Valley in the Sierra Nevada, Mayr and Armi (2010) found that foehn was only able to descend to the valley floor in the afternoon when diurnal heating had warmed the valley air mass sufficiently. These findings are consistent with observations from the Inn Valley located in the eastern European Alps where the foehn frequency peaks in the afternoon (Mayr *et al*., 2007).

*Process 2*. The transition zone between foehn flow and CAP is characterized by vertical wind shear. When the production of turbulence by wind shear is greater than its decay due to negative buoyancy and dissipation (i.e., Richardson number Ri below its critical value of 0.25), turbulent mixing between the foehn flow and the CAP below occurs, resulting in top‐down heating of the CAP. The turbulence in the transition zone is generated by Kelvin–Helmholtz (KH) instability (e.g., Fritts, 1982), which is one form of shear flow instability. KH billows and associated turbulence have been observed in several foehn studies (e.g., Nater *et al*., 1979; Attié *et al*., 1999; Lothon *et al*., 2003; Flamant *et al*., 2006; Strauss *et al*., 2016; Tollinger *et al*., 2019). However, for none of these observational studies the turbulent heat flux caused by shear flow instabilities was quantified. Thus, turbulent CAP erosion was studied based on theory and simulations rather than on observations. As mentioned above, a necessary condition for turbulent erosion is Ri<0.25. Based on this prerequisite, Petkovšek (1992) and Rakovec *et al*. (2002) found that wind speed above the CAP has to increase continuously in time to overcome the damping effect of the increasing stability at the top of the CAP. Using a semi‐analytical model, Zhong *et al*. (2003) argued that turbulent erosion is rather a slow process. Based on observations and simulations, Zhong *et al*. (2001) identified turbulent erosion as a minor process in the presence of a downslope windstorm above a stagnant CAP. They concluded that large negative buoyancy caused by a strong inversion at the top of the CAP completely counterbalanced shear‐induced turbulence. Conversely, based on a simulation of foehn in the Rhine Valley, Jaubert *et al*. (2005) found that turbulent erosion was a leading term in the heat budget of the CAP. Lareau and Horel (2015b) obtained similar results for idealized simulations. They found that advection terms in the heat budget had nearly compensating effects while turbulent heating led to top‐down dissipation of the CAP. Further, Fritts *et al*. (2010) identified KH instabilities to be of major importance in the removal of the Arizona Meteor Crater CAP. For two‐dimensional idealized simulations of a background flow past a CAP in a valley, Sheridan (2019) found that shear‐induced vertical mixing can play a crucial role in CAP erosion.

*Process 3*. For a case‐study in the Salt Lake Basin, Lareau and Horel (2015a) showed that CAP displacement by a mountain wave enabled the downslope windstorm to penetrate to the valley bottom. This interaction between mountain waves and the CAP in the Owens Valley was also investigated by Strauss *et al*. (2016) based on several cases from which four typical flow scenarios were derived. In one scenario, the valley air was flushed out by the downslope windstorm below a large‐amplitude gravity wave. However, they point out that, for this scenario, the valley atmosphere has to be well‐mixed, which points towards convection in the CAP discussed above (Process 1). Based on a case‐study of foehn in the Rhine Valley, Flamant *et al*. (2006) concluded that CAP displacement was more important for the CAP removal than convection inside and turbulent erosion at the top of the CAP.

In addition to the processes mentioned above, the outflow of the cold air from the valley due to a favourable pressure gradient has the potential to promote breakthrough of foehn at the valley floor (e.g., Zängl, 2005). For the Inn Valley, von Ficker (2012) suggested cold air drainage in the pre‐foehn stage is an important process which slows down the CAP's warming.

The interruption of foehn in a valley can be caused by nocturnal cooling and an associated reestablishment of the CAP which leads to lifting of the foehn flow from the valley floor and causes a minimum of foehn occurrence during night‐time in the Inn Valley (Mayr *et al*., 2007). For the Salt Lake Valley, Lareau and Horel (2015a) found that a downslope windstorm was interrupted by the CAP moving back after it was displaced locally. On the synoptic scale, foehn is often terminated by the passage of a cold front (e.g., Gohm *et al*., 2010). Further, cold‐air advection into the valley has been identified as a cause of foehn breakdown (Gohm *et al*., 2004; Zängl *et al*., 2004).

**Figure 1:**
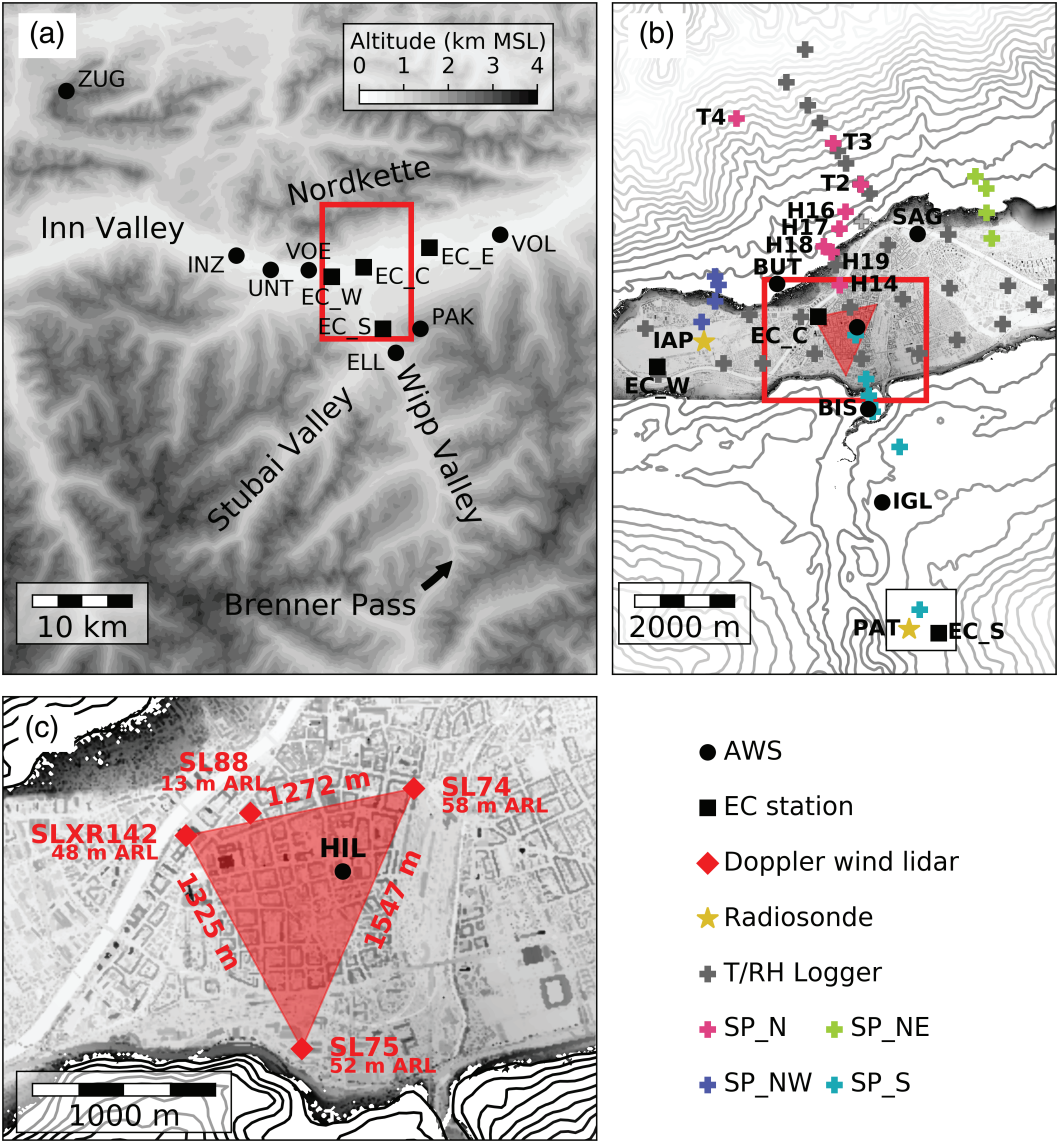
Target area and instrumentation of the PIANO field campaign. (a) Larger domain covering the east–west aligned Inn Valley in the vicinity of Innsbruck and the south–north orientated Wipp Valley with the Brenner Pass. (b) Close‐up of the city of Innsbruck at the exit of the Wipp Valley. (c) Doppler wind lidar arrangement in the city centre. Three of the lidars (SLXR142, SL75 and SL74) were installed on tall buildings forming a triangle. Their installation height above the reference level of 570 m MSL and the side lengths of the triangle are shown. The fourth lidar (SL88) was installed along the northern side of this triangle on a lower building. Terrain height is shown in (a) as grey shading and in (b) and (c) as contour lines with increments of 100 and 20 m, respectively, together with grey shaded building height. Markers indicate location of different instruments (see legend). The four slope profiles (SP_N, SP_NE, SP_NW and SP_S) of temperature and relative humidity (T/RH) loggers are marked with different colours. For SP_N stations, the abbreviation of each station used in this study is displayed. Area (b) is shown as a red box in (a), while the red box in (b) marks the subdomain (c)

The European Alps are a favourable mountain range for the occurrence of foehn. Mayr and Armi (2008) explain that foehn in the central Alps is a response to temperature differences in air masses between the upstream and downstream side. In the case that the upstream reservoir of cold air does not reach up to the crest height, flow is still possible through passes, which is known as shallow foehn in contrast to deep foehn. This increases the occurrence of foehn downstream of gaps in the main Alpine crest. One of the deepest Alpine gaps is the Brenner Pass (1371 m MSL). It represents the highest point of the north–south aligned Wipp Valley, which joins the east–west oriented Inn Valley at the city of Innsbruck (Figure 1a). When there is a CAP in the Inn Valley with foehn aloft, enhanced westerly (downvalley) winds with a maximum speed over the city have frequently been observed. These *pre‐foehn westerlies* are largest in the vicinity of Innsbruck. Based on model simulations, Zängl (2003) proposed that these pre‐foehn westerlies are a result of an asymmetry in gravity waves forming over the mountain ridges east and west of the exit of the Wipp Valley. This leads to an along‐valley pressure gradient near the surface with lower pressure to the east of Innsbruck. As a result, locally enhanced westerly low‐level winds form which are superimposed on a general, weak cold‐air outflow into the Alpine foreland (Zängl, 2003). For a case‐study, Zängl and Gohm (2006) found another mechanism which contributes to the evolution of the pre‐foehn westerlies: an eastward decreasing CAP depth intensifies the local surface pressure minimum and extends their occurrence eastwards.

In order to improve the understanding of the transient phases of foehn breakthrough and interruption in Alpine valleys, a measurement campaign was conducted in autumn and early winter 2017. This campaign is part of the research project Penetration and Interruption of Alpine Foehn (PIANO) and took place in the Wipp Valley and Inn Valley, Austria. These valleys have been a preferred study area for foehn research for several decades. In particular, in the framework of the Mesoscale Alpine Programme (MAP), new knowledge about foehn‐related phenomena was gained (e.g., Mayr *et al*., 2007). However, MAP and previous campaigns focused more on the well‐developed phase of foehn and less on the transient initial and final stages.

The role of turbulent CAP erosion in enabling foehn penetration is especially unclear. This is partly related to its small‐scale character which makes it difficult to observe and quantify. The measurement set‐up of the PIANO field campaign enables the assessment of turbulent erosion. Here, we analyse the second Intensive Observation Period of the PIANO campaign (PIANO IOP2), which took place from 3 to 5 November 2017. This event is characterized by more than 40 hr of continuous foehn in the Wipp Valley (with a transition from shallow to deep foehn) and different stages of foehn–CAP interaction in the Inn Valley. The goals of this work are to analyse the evolution leading to the final foehn breakthrough and interruption and also to identify the dominant processes of foehn–CAP interaction relevant for the CAP heat budget. In particular, this study aims to quantify the role of turbulent mixing based on observational data. The outline of this paper is as follows. The instrumental set‐up and the analysis methods are described in Section 2. The results are divided into two sections. First, an overview of the temporal evolution of the foehn event is given in Section 3. Second, different stages of this event are presented in more detail in Section 4. The results are discussed in Section 5 and the conclusions are drawn in Section 6.

## 2 DATA AND METHODS

### 2.1 Instrumentation

During the PIANO field experiment, the area around the city of Innsbruck was densely equipped with meteorological instruments. Besides the operational network of automatic weather stations (AWSs) of the Austrian national weather service ZAMG, nine portable AWSs, part of the so‐called mobile measuring network of Alpine atmospheric research (MOMAA), were operated (Figure 1a). Further, the Austrian air navigation service provider (Austro Control) and the federal state Tirol (Land Tirol) operate measurement stations in the target area. From the operational AWSs, we used temperature, humidity, pressure and wind data available at 10 min intervals. Temperature and humidity were typically measured at 2 m and wind at 10 m above ground level (AGL). In this work we will distinguish between height AGL, height above mean sea level (MSL) and height above reference level (ARL). The reference level always refers to 570 m MSL, which represents the approximate height of the Inn river in Innsbruck. Hence, for stations in Innsbruck, ARL and AGL are approximately the same. The MOMAA AWSs measured temperature and humidity at 2 m AGL (HC2A‐S3, Rotronic), horizontal wind speed and direction at 3.5 m AGL (WindSonic, Gill Instruments), and precipitation amount (tipping bucket rain gauge, Young) and air pressure at about 1 m AGL (CS100, Campbell Scientific). Data were stored at 1 min intervals. While the operational stations cover the mesoscale, the MOMAA AWSs increase the spatial coverage in the vicinity of Innsbruck. This network of AWSs was extended with four eddy‐covariance (EC) stations. Three of them were operated in the Inn Valley: in the centre of Innsbruck (EC_C), in the western part of Innsbruck near the airport (EC_W) and to the east of the city (EC_E). The fourth EC station was located in the Wipp Valley at the village of Patsch (EC_S). The EC stations were equipped with 3D sonic anemometers (CSAT3/CSAT3B, Campbell Scientific). They sampled wind and temperature at 10 Hz (EC_C) and 20 Hz (EC_E, EC_W and EC_S), from which we derived mean turbulent heat flux and turbulence kinetic energy (TKE) at 30 min intervals. Further, a temperature and humidity probe (HC2A‐S3, Rotronic) was mounted at each EC station. From those we used 1 min averaged temperature and humidity data. The temperature and humidity probes of EC_W, EC_E and EC_S were installed at about 2 m AGL above grassland and the 3D sonic anemometers at about 2.5 m AGL. The EC_C was located on a mast on a building, at about 43 m above street level. The density of meteorological measurements was highest in the city of Innsbruck (Figure 1b). In the target area, about 50 temperature and relative humidity (T/RH) loggers (HOBO MX2302A, Onset) were installed on a horizontal grid of about 1×1 km${}^{2}$ at the valley floor and along four slope profiles. The latter were located north (SP_N), northeast (SP_NE), northwest (SP_NW), and south (SP_S) of the city (Figure 1b). The T/RH loggers sampled instantaneous values of air temperature and relative humidity every minute. The SP_N profile was complemented with operational slope stations of the ZAMG (T2, T3, and T4 in Figure 1b). In contrast to other ZAMG AWSs, these stations are installed about 2 to 4 m AGL, e.g., on aerial lift pylons. During IOPs, radiosondes were launched regularly in the Wipp Valley, near the village of Patsch (PAT; 976 m MSL), and at Innsbruck airport (IAP; 579 m MSL). During PIANO IOP2, eleven radiosondes were launched in the Wipp Valley and twelve radiosondes at Innsbruck Airport.

Finally, four Doppler wind lidars (Stream Line, Halo Photonics) were operated within the city of Innsbruck (Figure 1c). Three were installed on the rooftop of tall buildings forming a triangle with side lengths of about 1,300 m (Figure 1c). The installation height was between 48 and 58 m ARL (Figure 1c). The fourth lidar was located on a lower building (13 m ARL) close to the northern triangle side. Three of them (SL74, SL75 and SL88) are Stream Line systems and the fourth lidar (SLXR142) is a Stream Line Extended Range (XR) unit. Each lidar has an all‐sky scanner that can be operated in continuous scanning mode (CSM), step‐and‐stare mode (SS) or in constant staring mode.

Doppler wind lidars measure the line‐of‐sight velocity, $v_{r}$, for a distance R away from the instrument. This line‐of‐sight velocity is the projection of the three‐dimensional wind vector, $u={\left(u,v,w\right)}^{T}$, on the lidar beam direction (e.g., Frehlich, 1997):

(1) $$v_{r}(R)=\hat{r}\cdot u(R)=u\cos el\sin az+v\cos el\cos az+w\sin el.$$

Here, $\hat{r}$ is the unit vector in direction of the lidar beam which is a function of the azimuth angle az and the elevation angle el. Our systems are pulsed Doppler wind lidars with pulse lengths of about 170 ns (SL74, SL75 and SL88) and 380 ns (SLXR142). The pulse repetition frequency is 15 kHz (SL74, SL75 and SL88) and 10 kHz (SLXR142). Profiles of radial velocity are derived for accumulated pulses resulting in different sampling frequencies. Hence, derived $v_{r}$ always represents a spatial and temporal average.

Since el and az are known parameters and $v_{r}$ is the measured quantity, Equation (1) is left with three unknowns, representing the three wind velocity components. This equation is the basis of several different scanning strategies and resulting retrievals. For a Doppler wind lidar operating in vertical stare mode ($el=90^{\circ }$), the vertical velocity, w, is measured directly. For a conical scan (e.g., CSM or SS scan with fixed elevation and equally spaced azimuth angles), the horizontal wind components can be estimated. With at least two Doppler wind lidars scanning along the same plane (i.e., coplanar scans), the two‐dimensional wind field on this plane can be retrieved. The algorithms used to derive various retrievals are described in Section 2.2.1.

**Figure 2:**
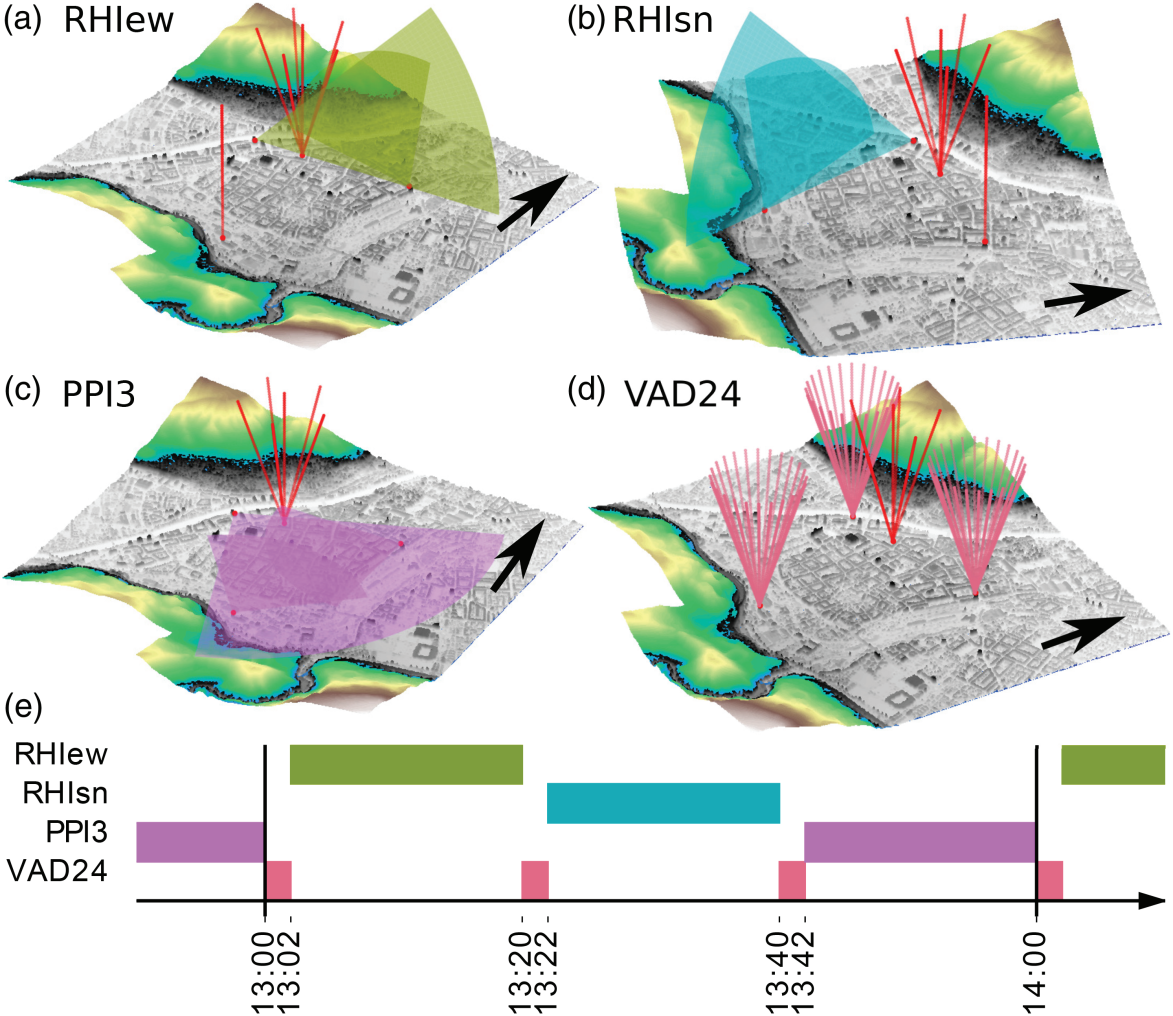
Doppler wind lidar scan pattern performed during the PIANO field experiment. (a)–(c) Coplanar scans with multiple lidars along different planes. Dual‐Doppler scans for (a) a vertical plane along the valley (RHIew) performed with the SLXR142 and SL74 lidar and (b) a vertical plane across the valley (RHIsn) performed with the SLXR142 and SL75 lidar, and (c) a nearly horizontal plane (PPI3) performed with the SLXR142, SL75 and SL74 lidar. (d) Conical scans with 24 beams (VAD24) performed with the SLXR142, SL75 and SL74 lidar simultaneously. In each panel, the black arrow points northward. (e) Example scan schedule for the period between 1300 and 1400 UTC. See Figure 1c for lidar locations and Table 1 for scan settings

Based on the triangle arrangement of the SL74, SL75 and SLXR142 (Figure 1c) we realized coplanar scans for two vertical and one horizontal plane. The vertical coplanar scans were conducted with two lidars performing range–height indicator (RHI) scans, whereas for the horizontal plane, all three lidars performed plan position indicator (PPI) scans. One vertical coplanar scan was performed with the SLXR142 and the SL74, approximately in the east–west direction along the Inn Valley (RHIew; Figure 2a). The second vertical coplanar scan was performed with the SLXR142 and SL75 approximately in an south–north direction across the Inn Valley towards the exit of the Wipp Valley (RHIsn; Figure 2b). The third lidar which was not involved in these vertical coplanar scans performed vertical stare measurements for this time period. The parameters of these three scan patterns are described in Table 1. For all scan patterns, we chose a range gate length of 18 m. The scanning sector and the rotation speed of the scanner were chosen such that each scan took approximately 30 s. This enabled the synchronization of the scans and led to a reduction of the error in the retrievals (Stawiarski *et al*., 2013). For the PPI scans, the constant elevation angle was non‐zero (PPI3; Figure 2c). This was necessary to prevent blocking of the laser beam by high buildings within the scanning area. Hence, the overlapping PPI cones of the three lidars are not perfectly coplanar and only nearly horizontal. However, the vertical distance from each other within the lidar triangles is less than 15 m.

**Table 1:** Lidar settings for the scan patterns RHIew, RHIsn and PPI3 (see Figure 2a–c)

Scan pattern	Parameters	SLXR142	SL74	SL75
RHIew	az / el (deg)	78.3 / 0–45	258.3 / 0–90	— / 90
	$v_{s}$ (deg·s${}^{-1}$) / $f_{s}$ (Hz)	1.5 / 2	3.0 / 2	0 / 1
RHIsn	az / el (deg)	151.4 / 0–45	— / 90	331.4 / 0–90
	$v_{s}$ (deg·s${}^{-1}$) / $f_{s}$ (Hz)	1.5 / 2	0 / 1	3.0 / 2
PPI3	az / el (deg)	70–160 /1.6	180–270 / 0.5	320–410* / 1.0
	$v_{s}$ (deg·s${}^{-1}$) / $f_{s}$ (Hz)	3.0 / 2	3.0 / 2	3.0 / 2

*Note*: For RHIew and RHIsn, two lidars performed coplanar scans on vertical planes in the east–west (RHIew) and north–south (RHIsn) directions, respectively, and the third lidar conducted vertical stare measurements. For the horizontal coplanar scan, PPI3, triple‐Doppler lidar scans were conducted at a fixed low elevation on a nearly horizontal plane. *corresponds to $az=50^{\circ }.$ Abbreviations: az=Azimuth angle; el=elevation angle; $f_{s}$=sampling frequency; PPI=plan position indicator; RHI=range–height indicator; $v_{s}$=rotation speed of scanner.

In order to capture the spatio‐temporal variation of the wind field for all three spatial dimensions during foehn events, the three coplanar scan patterns (RHIew, RHIsn, and PPI3) were consecutively performed within 1 hr (with a pause of 2 min between two patterns) before the whole sequence was repeated (Figure 2e). Therefore, each coplanar scan pattern covered a period of 18 min and consisted of 32 scans, each of which took about 30 s, as mentioned above. During the pause of 2 min, each of the three lidars (SL74, SL75 and SLXR142) performed a full‐rotation PPI scan in SS mode consisting of 24 equally spaced beams at a constant elevation angle of 70° (VAD24; Figure 2d). The fourth lidar (SL88) performed a so‐called six‐beam scan pattern (Sathe *et al*., 2015). For this purpose, a conical scan in SS mode with five equally spaced azimuth angles was performed, which was followed by a sixth beam pointing vertically. The whole pattern took about 26 s and was continuously repeated throughout the whole campaign.

### 2.2 Methods

#### 2.2.1 Doppler wind lidar retrievals

From the dataset of Doppler wind lidar measurements, a variety of parameters was retrieved: vertical profiles of the horizontal wind, fields of two‐dimensional wind in vertical and horizontal planes and vertical profiles of vertical velocity variances and spectra. In this section the algorithms for these retrievals are briefly described.

Vertical profiles of horizontal wind were calculated using lidar measurements performed along a cone (Section 2.1) by applying the Velocity–Azimuth Display (VAD) technique (Browning and Wexler, 1968). For the six‐beam scans conducted with SL88, the horizontal winds are not retrieved for single scans, but for averaging periods. For example, a 10 min averaged profile is calculated by collecting all beams on the cone (five per rotation) over a 10 min period (≈23 rotations) and applying the VAD algorithm. For each of the remaining three lidars, only a single full‐rotation based on 24 equally spaced beams was performed every 20 min. Hence, the resulting vertical profiles are averaged less in time but more in space than the SL88 profiles.

Algorithms for the calculation of the two‐dimensional wind field using coplanar lidar scans are presented in various studies (e.g., Newsom *et al*., 2008; Newsom *et al*., 2012; Stawiarski *et al*., 2013; Cherukuru *et al*., 2015; van Dooren *et al*., 2016). They are based on Equation (1). For horizontal scans, the elevation angle is set to zero ($el=0^{\circ }$) which simplifies Equation (1) from three to the two unknowns u and v:

(2) $$v_{r}(x,t)={\hat{r}}_{h}(t)\cdot v_{h}(x,t).$$

Here, ${\hat{r}}_{h}(t)={\left(\sin az,\cos az\right)}^{T}$ is the two‐dimensional unit vector in the direction of the horizontally pointing lidar beam and $v_{h}={\left(u,v\right)}^{T}$ represents the horizontal wind vector. For coplanar scans in a vertical plane, the azimuth angle is fixed. By introducing the variable $u_{v}=u\sin az+v\cos az$ (projection of $v_{h}$ on the vertical lidar plane), Equation (1) again reduces to two unknowns $u_{v}$ and w:

(3) $$v_{r}(x,t)={\hat{r}}_{v}(t)\cdot v_{v}(x,t),$$

with ${\hat{r}}_{v}(t)={\left(\cos el,\sin el\right)}^{T}$ being the two‐dimensional unit vector in the direction of the lidar beam and $v_{v}={\left(u_{v},w\right)}^{T}$ the two‐dimensional wind vector in the vertical plane. The procedure to derive $v_{h}(x,t)$ and $v_{v}(x,t)$ from radial velocity measurements is described in Appendix A.1. In contrast to former studies analysing coplanar scans of Doppler wind lidars (e.g., Newsom *et al*., 2008; Hill *et al*., 2010; Stawiarski *et al*., 2013), we used radial velocity measurements from three lidars (PPI3; Figure 2 and Table 1) to estimate the two‐dimensional wind field. The Python code of this algorithm can be found at Haid (2019).

The 1 Hz data of the vertical stare measurements are suitable for deriving vertical profiles of vertical wind velocity variances and spectra. They are available once per hour for 18 min periods. For each height within this time interval, the vertical velocity variance, $\bar{w^{\prime 2}}$, is derived. In a first step, each time series of vertical velocity is linearly detrended. The variances are then derived from those series with

(4) $$\bar{w^{\prime 2}}=\bar{{\left(w-\bar{w}\right)}^{2}}-\epsilon ,$$

where w are the detrended velocities and $\bar{w}$ their temporal mean. The variable ϵ represents the random error of the velocity estimator (Frehlich 1997; Appendix A.2). The detrended time series of vertical velocity are further used to estimate the power spectrum for each height. The frequency of the maximum in the power spectrum (i.e., the dominant frequency) can be interpreted as a characteristic time‐scale for turbulence production (Lothon *et al*., 2009).

#### 2.2.2 Potential temperature

The potential temperature used in this work is slightly different from the standard definition (AMS 2019) and represents the temperature which a parcel of air would reach if brought dry adiabatically to the reference level $z_{\text{REF}}=570$ m MSL For this procedure the dry adiabatic lapse rate $\gamma_{d}=0.0098$ K·m${}^{-1}$ is used. Hence, for a measured temperature T (K) at a level z (m MSL), the potential temperature θ is given by

(5) $$\theta =T+\gamma_{d}(z-z_{\text{REF}}).$$

This definition is more convenient for the analysis of temperature from stations that do not record atmospheric pressure.

#### 2.2.3 Richardson number

The Richardson number is an important parameter used to estimate whether the state of the atmosphere enables the production of turbulence. It is a dimensionless ratio of buoyant suppression of turbulence to shear generation of turbulence (AMS, 2019). For measurements at discrete measurement levels, the Richardson number can be approximated by the bulk Richardson number, $R\;i_{b}$:

(6) $${Ri}_{b}=\frac{\frac{g}{\theta_{0}}\frac{\Delta \theta }{\Delta z}}{{\left(\frac{\Delta u}{\Delta z}\right)}^{2}+{\left(\frac{\Delta v}{\Delta z}\right)}^{2}}.$$

Here, g is the acceleration due to gravity and $\theta_{0}$ the mean potential temperature of the considered layer. The change of potential temperature with height can be estimated from the slope profile SP_N (Figure 1b) and the profiles of the wind components, u and v, can be retrieved from data of the SL88 lidar. Here, we used SP_N because it was located closest to the SL88 lidar and had the greatest vertical extent (Figure 1b). The vertical resolution of the temperature slope profile is coarser (about 50 to 350 m) than the wind profile (18 m), i.e., Δz in Equation (6) differs for temperature and wind gradients. Hence, for calculating $R\;i_{b}$ at a certain lidar level, we use the corresponding mean temperature gradient over a deeper layer.

## 3 TEMPORAL EVOLUTION OF THE FOEHN EVENT

### 3.1 Synoptic and mesoscale overview

**Figure 3:**
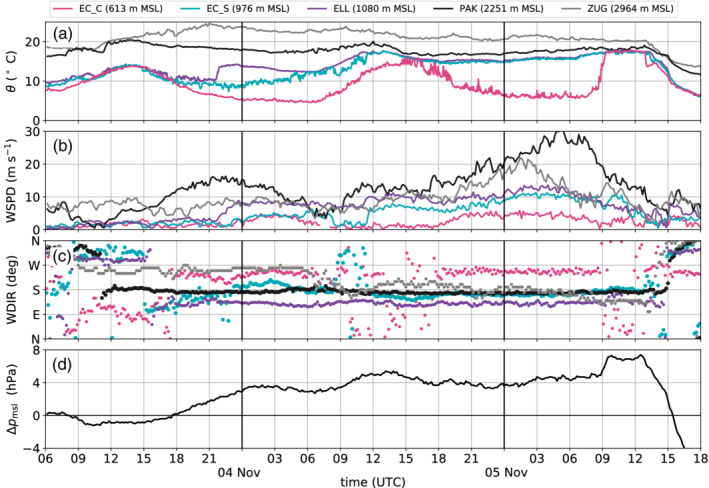
Measurements of (a) potential temperature θ, (b) wind speed WSPD, and (c) wind direction WDIR, from five weather stations between 0600 UTC on 3 November and 1800 UTC on 5 November 2017. Patscherkofel (PAK; 2,251 m MSL) and Zugspitze (ZUG; 2,967 m MSL) are mountain stations, Ellboegen (ELL; 1,080 m MSL) and EC_S (976 m MSL) are located in the Wipp Valley, and EC_C (613 m MSL) is located in the centre of the Inn Valley (Figure 1a). (d) Difference in mean sea level pressure between Bolzano (south of the main Alpine crest) and Innsbruck ($p_{\text{msl}}(\text{Bolzano})-p_{\text{msl}}(\text{Innsbruck})$)

**Figure 4:**
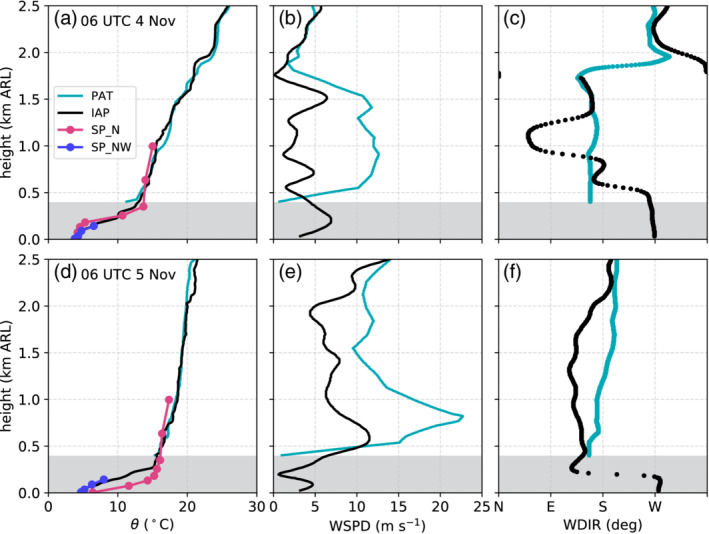
Vertical profiles for (a)–(c) 0600 UTC on 4 November and (d)–(f) 0600 UTC on 5 November 2017 of (a, d) potential temperature θ, (b, e) wind speed WSPD, and (c, f) wind direction WDIR above the reference level (570 m MSL) based on radiosonde ascents at Patsch (PAT) and Innsbruck Airport (IAP) as well as two slope profiles SP_N and SP_NW. The grey shaded layer indicates the depth of the Inn Valley below the height of Patsch

On 3 November 2017 the Alps were under the influence of a cut‐off low centred northwest of Spain. This situation led to the advection of potentially warmer air to the northern side of the Alps, building up a cross‐Alpine pressure gradient (Figure 3d). As a result, flow over the Brenner Pass initiated foehn in the Wipp Valley (Figure 1a). The onset of foehn was first observed at the mountain station Patscherkofel (PAK; 2,251 m MSL) south of Innsbruck at 1100 UTC on 3 November 2017 (in the target area, the local time=UTC+1 hr). At that time PAK measured a sudden warming, accompanied by a wind shift from northwesterly to southerly directions and an increase in wind speed (Figure 3a–c). The mountain station Zugspitze (ZUG; 2,964 m MSL), Germany, 50 km northwest of Innsbruck, observed moderate westerly winds. Considering ZUG being representative for the large‐scale flow at crest height, this event started as a *shallow foehn* (e.g., Zängl, 2003; Gohm and Mayr, 2004). Radiosondes launched at the village of Patsch (PAT; 976 m MSL) and at Innsbruck Airport (IAP; 579 m MSL) at 0600 UTC on 4 November (Figure 4a–c) confirm the shift to westerlies above crest level (∼1,800 m ARL; Figure 4c). At 1800 UTC on 3 November the pressure difference between Innsbruck and a station south of the main Alpine crest (Bolzano; 262m MSL) became positive (Figure 3d) and by 2200 UTC the foehn had advanced along the Wipp Valley to the station Ellboegen (ELL; 1,080 m MSL) which is located 20 km downvalley of the Brenner Pass. The foehn breakthrough at ELL was characterized by a jump in temperature and an increase in wind speed (Figure 3a,b). About 5 km further north at Patsch, the onset of foehn was a gradual process, as illustrated by a more continuous rather than abrupt temperature increase during the night (EC_S in Figure 3a). During this pre‐foehn stage, the surface flow at Patsch had a westerly component (Figure 3c), suggesting that the station might have been affected by the outflow from the adjacent Stubai Valley (Figure 1a). From about 0500 UTC on 4 November onwards, foehn air was present at Patsch, as seen in the potential temperature convergence between ELL and EC_S (Figure 3a) and a turn in wind direction to south at EC_S (Figure 3c). Interestingly, there was a period of foehn interruption at Patsch between about 0800 and 1100 UTC on 4 November when winds were weaker, the wind direction fluctuated between north and south (Figure 3b,c) and potential temperatures were lower than at ELL (Figure 3a). During this time, Patsch was most likely affected by the cold pool that temporarily spread from the Inn Valley into the Wipp Valley.

During the night between 3 and 4 November 2017, potential temperatures were about 4 to 5 K higher at PAK than at ELL, i.e., the foehn flow in the Wipp Valley was not well mixed (Figure 3a). This stable stratification of the foehn flow is also visible in the vertical profiles of potential temperature recorded by the radiosondes launched at 0600 UTC on 4 November at PAT and IAP. Both show an average vertical potential temperature gradient of about 5.4 K·km${}^{-1}$ (Figure 4a). The foehn flow in the Wipp Valley at PAT was characterized by an approximately 1,400 m deep southerly jet with horizontal wind speeds up to about 12 m·s${}^{-1}$ (Figure 4b,c). At that time, surface winds at PAT were not very strong (5 to 6 m·s${}^{-1}$; EC_S in Figure 3b) but potential temperature was the same as at ELL (Figure 3a). In the Inn Valley a CAP was present in the lowest 400 m above the valley floor (Figure 4a) with pre‐foehn westerlies (Figure 4b,c). Above the CAP, the foehn flow was weaker than in the Wipp Valley with partly reversed (northerly) components (IAP; Figure 4b,c) which could be a sign of wave breaking and/or flow splitting at the mountain range north of Innsbruck.

During 4 November, the cut‐off low merged with an eastward moving mid‐tropospheric pressure trough which turned the synoptic‐scale flow over the Alps to a southwesterly direction and later on 4 November, with the trough moving further east, to a southerly direction. This change in the large‐scale flow direction was accompanied by a further increase in the cross‐Alpine pressure difference (Figure 3d) and a wind shift at ZUG from westerly to southerly directions between 0600 and 0900 UTC on 4 November (Figure 3c). This wind shift marks the transition from *shallow* to *deep foehn*. The latter is supported by southerly flow above crest level (about 2,000 m ARL) in the two soundings conducted at 0600 UTC on 5 November (Figure 4f). At PAK this transition was displayed as a steady increase in wind speed after 0900 UTC on 4 November (Figure 3b). The static stability of the foehn flow decreased with its deepening, resulting in a weaker mean vertical gradient of potential temperature of 2.8 K·km${}^{-1}$ at 0600 UTC on 5 November (Figure 4d) and a reduced potential temperature difference between PAK and ELL of about 1.7 K (Figure 3a).

A sudden increase in potential temperature and a change in wind direction at the eddy covariance station in the centre of Innsbruck around 0900 UTC on 5 November indicated the penetration of foehn into the Inn Valley at this location (EC_C in Figure 3a). The station remained within foehn air for about 4 hr. Interestingly, breakthrough occurred during decreasing wind speed in the Wipp Valley (ELL and EC_S) and at the mountain peaks (PAK and ZUG). In the afternoon of 5 November, a cold front passed the Alps and terminated the foehn event. The mountain stations ZUG and PAK recorded the frontal passage at 1400 and 1500 UTC, respectively, with a drop in temperature and a wind shift to a northerly direction (Figure 3a,c). The stations in the Wipp Valley reported this wind shift slightly prior to the mountain station PAK (Figure 3c), which is the result of the typical backward inclination of the cold front surface relative to the direction of propagation (Gohm *et al*., 2010).

### 3.2 Evolution in the Inn Valley

**Figure 5:**
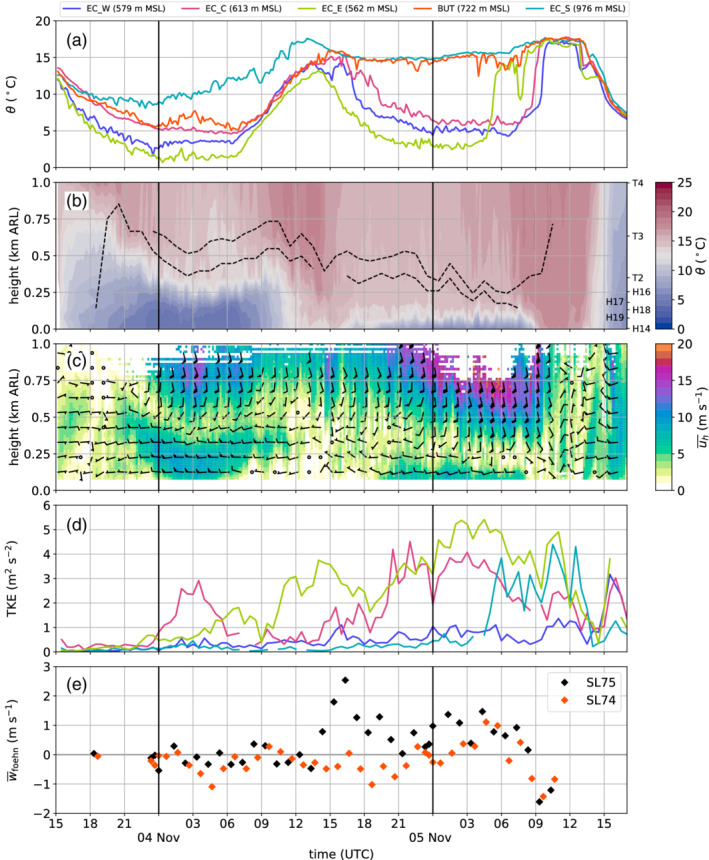
Time series of various quantities measured primarily in the Inn Valley between 1500 UTC on 3 November and 1700 UTC on 5 November 2017. (a) Potential temperature θ at the four EC stations and on the slope north of Innsbruck (BUT). (b) Time–height diagram of potential temperatures as colour shading (1 K intervals) measured by T/RH loggers along the northern slope of the Inn Valley (SP_N). Slope stations are indicated on the right axis. The dashed black lines mark the top of the pre‐foehn westerlies and the lowest height of the southerly foehn flow. Both are determined from one‐hourly averaged horizontal winds measured with the SL88 lidar (Section 2.2.1). The bottom of the foehn flow (top of the pre‐foehn westerlies) is the minimum (maximum) height for which the wind direction is $180\pm 20^{\circ }$ ($270\pm 20^{\circ }$) and the wind speed exceeds 1 m·s${}^{-1}$. (c) Time–height diagram of 10 min averaged horizontal winds (colour shading for speed ${\bar{u}}_{h}$ with intervals of 1 m·s${}^{-1}$ and barbs for horizontal direction and magnitude) measured with the SL88 lidar. Half barb, full barb, and triangle denote 2.5, 5, and 25 m·s${}^{-1}$. Heights in (b) and (c) are relative to the reference level of 570 m MSL (d) Turbulence kinetic energy (TKE) derived for 30 min intervals at the four EC stations. (e) Mean vertical wind speed ${\bar{w}}_{\mathrm{fohn}}$ within the foehn flow (above the upper black dashed line in (b)) measured with the lidars SL75 and SL74. Data are averaged over an 18 min interval, when lidars operated in vertical stare mode (Table 1). Figure 1 shows station locations

An overview of the evolution in the Inn Valley is gained by examining the time series of potential temperatures for selected weather stations. EC_E, EC_W and EC_C represent locations at the bottom of the Inn Valley east, west and in the centre of Innsbruck, respectively (Figure 5a). Buttererbichl (BUT; 722 m MSL) is located on the slope north of the city at about 150 m ARL. The station close to Patsch in the Wipp Valley (EC_S; 976 m MSL) is added in Figure 5a as a reference for the foehn temperature. However, notice that before 1100 UTC on 4 November, Patsch was in a transient state with from time to time lower potential temperatures than at Ellboegen (Section 3.1 and Figure 3a). In the afternoon of 3 November until 0000 UTC on 4 November, the temperatures in the valley decreased continuously and a stably stratified CAP formed. This CAP formation is also depicted by potential temperatures of the slope profile SP_N (Figure 5b). During the second half of the night, the CAP had a nearly constant depth of about 300 to 400 m which is supported by the morning sounding shown in Figure 4a. Furthermore, Figure 4a illustrates excellent agreement between the radiosonde and the two slope profiles.

While potential temperatures decreased in the lowest 300 m above the valley floor during CAP formation in the first half of the night from 3 to 4 November, the slope stations above observed increasing temperatures (Figure 5b) as a result of the strengthening of the south foehn aloft (Figure 5c). For example, a sudden temperature increase at 2230 UTC on 3 November at slope station T2 (350 m ARL; Figure 5b) was caused by the subsidence of the southerly foehn flow to this level above Innsbruck (Figure 5c), which was also the lowest foehn level during the rest of the night. Further below in the CAP, pre‐foehn westerlies of 6 to 10 m·s${}^{-1}$ prevailed until about 1200 UTC on 4 November (Figure 5c). During the first night, the CAP had a two‐layer structure with an inversion at its top and a less stable layer underneath (Figure 4a).

After 0000 UTC on 4 November, the temperatures in the vicinity of Innsbruck remained nearly constant until about sunrise at 0600 UTC on 4 November (EC_W, EC_C and EC_E in Figure 5a), whereas they further decreased during the night in the western (upper) Inn Valley where the valley atmosphere was more sheltered and presumably less affected by the foehn aloft (not shown). The lowest level of the foehn flow descended to about 500 m ARL at 0300 UTC on 4 November before rising again to about 700 m ARL (Figure 5b). At the same time the station BUT recorded an increase in potential temperature with a peak at 0300 UTC on 4 November (Figure 5a). The transition zone between pre‐foehn westerlies in the CAP and the south foehn aloft was about 150 m deep during the first night, and is marked by two dashed lines in Figure 5b. The lower line indicates the top of the pre‐foehn westerlies and the upper line represents the lowest foehn layer. The transition layer between these two heights is characterized by strong directional wind shear, weak mean horizontal wind speeds near the centre and a near‐neutral stratification, which are signs of strong turbulent mixing (Figure 5b,c). Similar characteristics were also observed by the radiosonde launched at 0600 UTC on 4 November at IAP (Figure 4a–c).

After sunrise at about 0600 UTC on 4 November, the potential temperatures at all stations started to increase (Figure 5a) and the stable stratification of the CAP weakened (Figure 5b). Simultaneously, the pre‐foehn westerlies weakened near the surface but prevailed until about 1400 UTC in the upper part of the CAP (Figure 5c). Despite the warming after sunrise, a potential temperature difference between the foehn air (EC_S) and the air near the valley floor (EC_C) of about 5 K remained throughout the morning (Figure 5a). Between 1100 and 1500 UTC, the bottom of the foehn flow descended again to about 500 m ARL (Figure 5b,c). At the end of this subsidence period, foehn broke through at the slope station BUT (150 m ARL) and remained until the end of the IOP, apart from a few short interruptions characterized by sudden cooling below foehn temperature (cf. BUT and EC_S in Figure 5a). After the foehn breakthrough at BUT until about 1900 UTC, the stations in the centre (EC_C) and west (EC_W) of the city experienced transient periods of warming, nearly reaching foehn temperature, as a result of downward transport and mixing of foehn air into the CAP. This almost complete foehn breakthrough is also illustrated by the weak stratification along the northern slope profile (Figure 5b). In contrast, the near‐surface air east of the city (EC_E) was unaffected by this warming, but continuously cooled in the afternoon to form a night‐time CAP. The same behaviour was observed at other stations east of the city, e.g., VOL, and west of the airport, e.g., VOE (not shown). After the phase of transient warming, a CAP formed also in the centre and western part of the city, although with 2 to 4 K higher temperatures than in the east (Figure 5a). However, with a depth of about 200 m in the centre of the city, the CAP was shallower during the second night than in the first night of this IOP (Figures 4a,d and 5b). During the second night, the bottom of the southerly foehn flow continuously descended (Figure 5b). On average, the transition zone between CAP and foehn was 150 m thick and was again characterized by near‐neutral stratification (Figure 5b).

In the morning of 5 November 2017, foehn broke through first in the eastern part of the city with an abrupt temperature increase before sunrise at about 0530 UTC (EC_E in Figure 5a). It took about another 3.5 to 4 hr until foehn established in the remaining parts of Innsbruck, first over the city centre (EC_C) and afterwards west of the city (EC_W). Henceforth, we call this incident at about 0900 UTC, when most stations around Innsbruck recorded the onset of the last foehn period of this event, the *final breakthrough* of IOP2. Surprisingly, the wind profiles show a strong deceleration of the foehn flow over the city centre during the time of the breakthrough and afterwards (Figure 5c). During this period, the wind direction was highly variably with even northerly (reversed) flow between 1100 and 1200 UTC.

**Figure 6:**
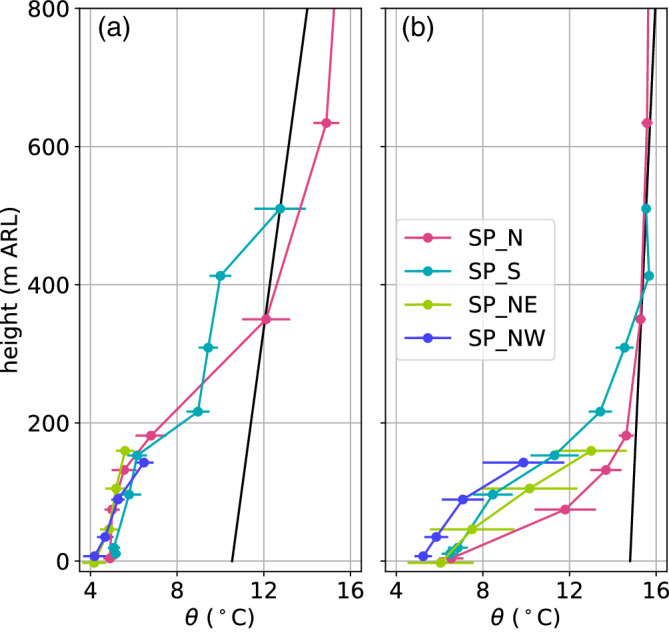
(a) Pseudo‐vertical profiles of potential temperatures θ averaged between 2000 UTC on 3 November and 0600 UTC on 4 November 2017 along four different slopes (cf. Figure 1b). The potential temperatures are calculated from the T/RH logger measurements according to Equation (5). The horizontal bars represent the temperature fluctuations during the observed period. These fluctuations are computed by subtracting the linear trend from each time series and calculating their standard deviation. (b) is as (a) but for the period 2000 UTC on 4 November to 0600 UTC on 5 November. Black straight lines in (a) and (b) represent extrapolated temperature profiles based on the mean stratification of the foehn layer between ELL and PAK. The highest level of the SP_S profile represent the station ELL in the Wipp Valley (Figure 1a)

The four slope profiles within the Inn Valley enable characterization of the spatial variability of the CAP. For this purpose, 10 hr averaged profiles are analysed for each of the nights: between 2000 UTC on 3 November and 0600 UTC on 4 November (Figure 6a) and from 2000 UTC on 4 November to 0600 UTC on 5 November (Figure 6b). Horizontal bars represent the standard deviation of the linearly detrended time series of potential temperature. In order to estimate whether temperatures at a slope station are below foehn temperature, two extrapolated temperature profiles are drawn in Figure 6 for each night based on the average stratification of the foehn flow in the Wipp Valley derived from the potential temperature gradient between ELL and the mountain station PAK. Comparisons with radiosonde data showed that this is a good approximation of the night‐time stratification of the foehn layer above Innsbruck (not shown). As already mentioned in Section 3.1, the foehn layer was more stable in the first IOP night (cf. extrapolated profiles in Figure 6a,b).

During the first night (Figure 6a), the four slope profiles look very similar in the lowest 150 m. The stability within the CAP in terms of potential temperature gradient is approximately 1 K·(100 m)${}^{-1}$. Due to the lower vertical extent of the two profiles SP_NE and SP_NW, the capping inversion of the CAP is only visible in the southern (SP_S) and northern (SP_N) profile. Above 200 m ARL, the northern profile becomes warmer than the southern profile. This difference indicates that the depth of the upper part of the CAP (above 200 m ARL) was not constant but decreased across the valley from south to north. This asymmetry is supported by larger temperature fluctuations in the northern profile at about 350 m ARL (standard deviations of about 0.6 K in Figure 6a). It was already mentioned that the sensor at this height (T2) recorded a sudden jump to foehn temperature during the night, resulting in a large standard deviation despite linear detrending.

In the second night of IOP2 (Figure 6b), the differences of the four mean slope profiles in the lowest 200 m were much larger. The lowest temperatures were measured at SP_NW, followed by SP_S, SP_NE and SP_N. The depth of the CAP can be estimated from the height where each of the profiles reach the extrapolated foehn temperature (black line in Figure 6b). This depth is about 200 m at SP_N and about 350 m at SP_S. Hence, the CAP is shallower to the north than to the south of the city, and therefore the top of the CAP is tilted across the valley. The full depth of the CAP cannot be estimated for the profiles SP_NW and SP_NE since they do not reach high enough to capture the near‐neutral foehn flow. Nevertheless, from comparing the mean CAP temperatures of SP_NE and SP_NW, it is conceivable that the CAP is slightly shallower in the eastern than in the western part of the city, but even shallower between, i.e., north of the city (SP_N). Hence, the CAP structure in Innsbruck is heterogeneous in both cross‐ and along‐valley directions. The lower depth of the CAP in the second night is consistent with higher static stability. The mean potential temperature gradient is about 3 to 4 K·(100 m)${}^{-1}$. Standard deviations of the linearly detrended time series of potential temperature in the CAP are up to 2.2 K. This is much larger than the first night and the foehn flow aloft, and is caused by temporal variability in the CAP's depth and frequent intrusions of foehn air (Figure 6a,b).

## 4 DISTINCT PHASES AND PROCESSES OF FOEHN–CAP INTERACTION

IOP2 passed through different stages characterized by a variety of flow features and processes. The most prominent phases of the foehn event will be presented in chronological order in this section and afterwards discussed in Section 5. Altogether six phases are distinguished: regular oscillations within the cold pool from 3 to 4 November (Section 4.1), transient breakthrough of foehn in the afternoon of 4 November (Section 4.2), shear flow instabilities in the night from 4 to 5 November (Section 4.3), the final breakthrough on 5 November (Section 4.4) and rotor formation above the Inn Valley (Section 4.5) before the foehn breakdown (Section 4.6).

### 4.1 Oscillations in the cold‐air pool

During the first night of IOP2 (3 to 4 November 2017), regular up‐ and downdraughts were detected by the vertical stare measurements of the Doppler wind lidars. From 1900 UTC on 3 November onwards, these oscillations were visible at all four lidar sites for a period of about 6 hr. Thus, they were present during the CAP formation in the Inn Valley as well as during the descent of the foehn flow to a level of about 500 m ARL (Figure 5b,c).

**Figure 7:**
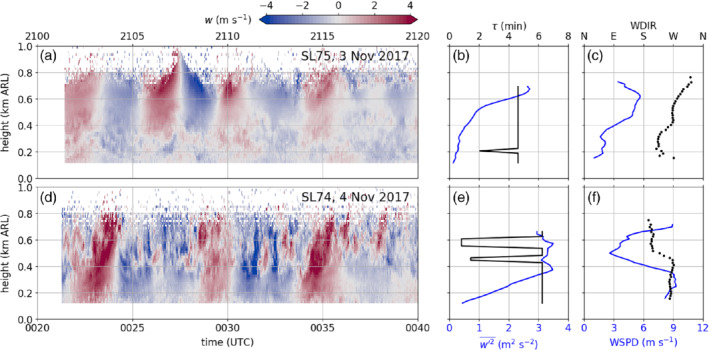
(a) Time–height diagram of vertical velocities observed with the SL75 lidar between 2102 and 2120 UTC on 3 November. Positive (negative) values refer to updraughts (downdraughts). (b) Profile of vertical velocity variance ($\bar{w^{\prime 2}}$, blue line) and profile of the period corresponding to the dominant frequency of the power spectrum of vertical velocity (τ, black line). (c) Averaged profiles of horizontal wind speed (WSPD, blue line) and direction (WDIR, black dots) measured at the beginning and end of the shown period. The profiles are retrieved from single PPI scans performed with the particular lidar (Section 2.1). (d, e, f) are as (a, b, c) respectively, but for the SL74 lidar between 0022 and 0040 UTC on 4 November 2017

The oscillations occurred first at the highest detectable altitude (∼800 m ARL) at about 1900 UTC on 3 November and later expanded through the whole CAP. An example is shown in Figure 7a for an 18 min period after 2102 UTC measured with the SL75 lidar. At this time the regular up‐ and downdraughts are mainly visible between 400 and 800 m ARL. The vertical velocity field below 400 m is characterized by less‐organized small‐scale motions. The height of this transition zone coincides with a change in wind speed and wind direction (Figure 7c). The lower layer is characterized by weak southwesterly flow, whereas in the upper layer moderate pre‐foehn westerlies prevail. For this 18 min time frame, the dominant period of the oscillations, τ, and the vertical velocity variance are derived as described in Section 2.2.1 and shown as vertical profiles in Figure 7b. At essentially all levels τ=4.6 min, even in the layer below 400 m ARL. The vertical velocity variance is below 1 m${}^{2}\cdot$s${}^{-2}$ in the lower layer, but increases above 2.5 m${}^{2}\cdot$s${}^{-2}$ in the upper layer.

Until midnight, the CAP with pre‐foehn westerlies became shallower (Figure 5c). Between 0022 and 0040 UTC on 4 November, the transition zone between the westerlies and the foehn was located between 400 and 500 m ARL and was characterized by both directional and speed shear (Figure 7f). In this transition layer, regular up‐ and downdraughts were superimposed by higher‐frequency fluctuations (Figure 7d). These fluctuations indicate stronger turbulence and vertical velocity variances exceeding 3 m${}^{2}\cdot$s${}^{-2}$ in this layer (Figure 7e). In the CAP the dominant period of the oscillation is about 6.2 min (Figure 7e). However, spectral analysis for the transition layer above has shown that the power spectrum does not have one single peak, but rather two peaks due to the higher‐frequency fluctuations (not shown). This results in jumps between two dominant periods of about 1 and 6.2 min within this layer (Figure 7e).

**Figure 8:**
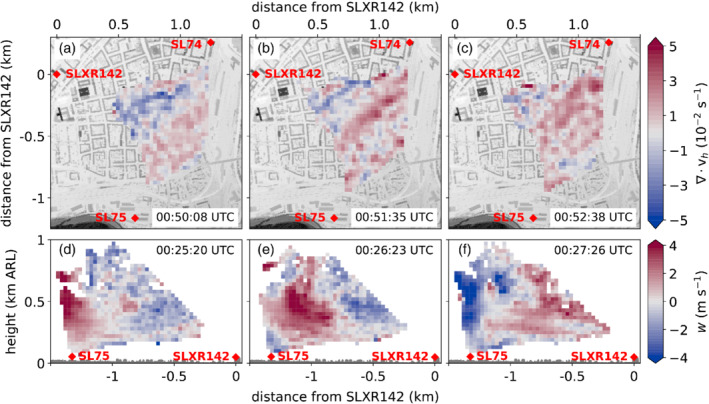
Smoothed divergence of the horizontal wind, $\nabla \cdot v_{h}$, at about 60 m ARL derived from nearly horizontal coplanar scans (PPI3; Table 1 and Figure 2c) at (a) 0050:08 UTC, (b) 0051:35 UTC and (c) 0052:38 UTC on 4 November 2017. Vertical wind velocity, w, derived on a nearly south–north oriented vertical plane between SL75 and SLXR142 (RHIsn; Table 1 and Figure 2b) at (d) 0025:20 UTC, (e) 0026:23 UTC and (f) 0027:26 UTC on 4 November. In (a)–(c) the building height is shaded. In (d)–(f) the height of the buildings along the transect is indicated in dark grey. The times given on each panel represent the starting times of the corresponding scan

In order to gain more details about the spatial structure of these oscillations and the propagation characteristics, coplanar retrievals were analysed. Throughout the 6 hr period from 1900 UTC on 3 November to 0100 UTC on 4 November, the horizontal wind field was characterized by elongated bands of alternating convergence and divergence propagating through the measurement area. Figure 8a–c shows an example of the smoothed horizontal wind divergence $\nabla \cdot v_{h}$ for three consecutive times. In order to make the convergence/divergence patterns easier to identify, the two‐dimensional divergence fields are smoothed using the smooth–desmooth operator of Shapiro (1970). In the example shown, a southwest‐to‐northeast divergence band (positive values) propagates perpendicular to its orientation across the city centre in a northwesterly direction. Low‐level horizontal divergence (convergence) is associated with negative (positive) vertical velocities. Such corresponding up‐ and downdraughts are shown in Figure 8d–f for three consecutive coplanar scans conducted with the lidars SL75 and SLXR142 across the valley approximately half an hour prior to the shown divergence fields. Consistent with the convergence field, these alternating up‐ and downdraughts propagate northward across the city.

The orientation and propagation of the observed wave patterns were derived quantitatively from cross‐correlation of vertical velocities measured simultaneously with all four lidars between 2300 UTC on 3 November and 0000 UTC on 4 November. The temporal shift for which the cross‐correlation coefficient reaches a maximum can be interpreted as the time needed for the wave signal to propagate from one lidar site to another. Since the wave signal propagates approximately from south to north (cf. Figure 8), the time series of the southernmost lidar (SL75) was used as the reference: its time series was correlated with the time series of the other three lidars (time series averaged vertically between 200 and 500 m ARL). For the correlation with the lower‐resolution SL88 time series, the reference was sub‐sampled to the corresponding lower interval of about 26 s. The time lag is smallest (3 min 6 s) for the SL74 lidar northeast of the SL75, followed by 3 min 20 sec for the SL88 lidar north of the SL75, and 3 min 34 sec for the SLXR142 lidar northwest of the SL75. These three time lags correspond to a phase line orientation of about 72° (southwest to northeast) and a phase speed of about 6.3 m·s${}^{-1}$ (northwestward propagation towards an azimuth angle of 342°). The wavelength can be estimated using the period, τ, of the vertical velocity oscillation and the phase speed of the waves. For the SL74 lidar, τ=255 s on average between 2000 UTC on 4 November and 0100 UTC on 5 November. Multiplied by the propagation speed 6.3 m·s${}^{-1}$, a wavelength of ∼1,600 m is deduced. Since this is larger than the coverage of the coplanar retrievals (Figure 8), the wavelength cannot be deduced directly from our lidar scans (PPI and RHI). However, using only measurements from the PPI scans of the SLXR142 lidar, which have a larger range of about 1.5 km, the wavelength can be estimated by fitting a sine wave through the measured radial velocity fluctuations. For the period between 2000 UTC on 4 November to 0100 UTC on 5 November, this leads to an average wavelength of ∼1,300 m. Given the differences in the calculation method (e.g., phase speed derived only for a 1 hr period), the agreement of these two mean wavelength estimates is satisfying.

### 4.2 Partial breakthrough

**Figure 9:**
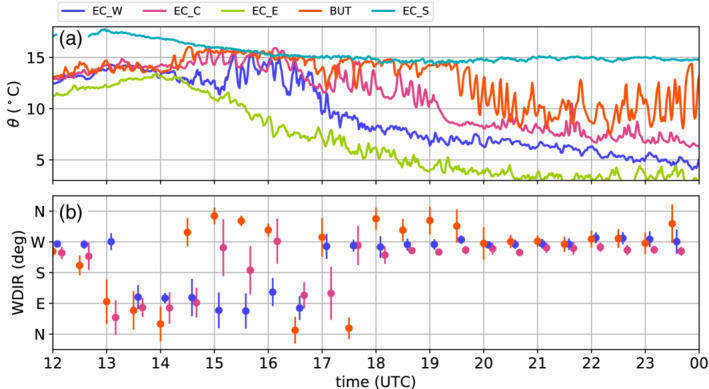
(a) Potential temperatures θ, measured near Innsbruck airport (EC_W), in the city centre (EC_C), about 6 km east of the city centre (EC_E), and on the slope north of the city (BUT; 152m ARL) between 1200 UTC on 4 November and 0000 UTC on 5 November 2017. Potential temperatures at EC_S (Wipp Valley) are added as a reference station for foehn temperature. (b) Half‐hourly averaged wind direction and standard deviation for the stations EC_W, EC_C and BUT

In the afternoon of 4 November 2017, stations in the centre and to the west of Innsbruck observed a period of intermittent foehn. In the observations close to the valley bottom, this is reflected by fluctuations in potential temperature with their maxima reaching the foehn temperature (EC_W and EC_C in Figure 9a). These fluctuations have a period of several minutes and amplitudes of up to 4 K. At a slope station north of Innsbruck (BUT; 152 m ARL), foehn was less intermittent and lasted until the evening (Figure 9a). In contrast, the potential temperature evolution east of Innsbruck followed a more typical (non‐foehn) diurnal cycle with a continuous decrease in the afternoon (EC_E in Figure 9a).

Shortly after 1400 UTC, the station BUT recorded the onset of foehn with a sudden temperature increase. Winds during foehn were from the northwest (Figure 9b), indicating reversed flow from the mountain ridge north of Innsbruck (Nordkette; Figure 1a). This suggests that the foehn air at this station originated from a foehn branch deflected at the Nordkette. Simultaneously with the onset of foehn, the SL75 lidar, located close to the exit of the Wipp Valley, measured a strong increase in mean vertical velocity within the foehn flow from ±0.4 m·s${}^{-1}$ up to 2.9 m·s${}^{-1}$ (Figure 5e). This indicates a change in the flow structure above the CAP. The next station observing foehn was EC_C (Figure 9a), followed half an hour later by HIL located further south (not shown). At both stations (HIL not shown), the wind direction was variable during foehn breakthrough, and hence did not reveal a clear direction from which the foehn flow penetrated to the valley floor (Figure 9b). After 1500 UTC on 4 November, fluctuations started also at EC_W accompanied by easterly flow. After sunset at 1600 UTC on 4 November, a stable stratification started to form in the Inn Valley and pre‐foehn westerlies re‐established (Figure 5c). These pre‐foehn westerlies advected potentially colder air from the upper Inn Valley towards the city. In the west of Innsbruck, this cold air advection led to a termination of the foehn at about 1700 UTC. Despite these westerly winds also being present at EC_C and HIL between 1700 and 1930 UTC, fluctuations in potential temperature continued (Figure 9a,b). For this period, BUT remained in foehn air and continued to observe northwesterly flow.

After 1930 UTC, all stations were located in the CAP and recorded pre‐foehn westerlies (Figure 9a,b). However, the large fluctuations in the temperature series of BUT suggest that this station was located at the interaction zone between the CAP and the foehn flow throughout the night.

### 4.3 Shear flow instabilities

**Figure 10:**
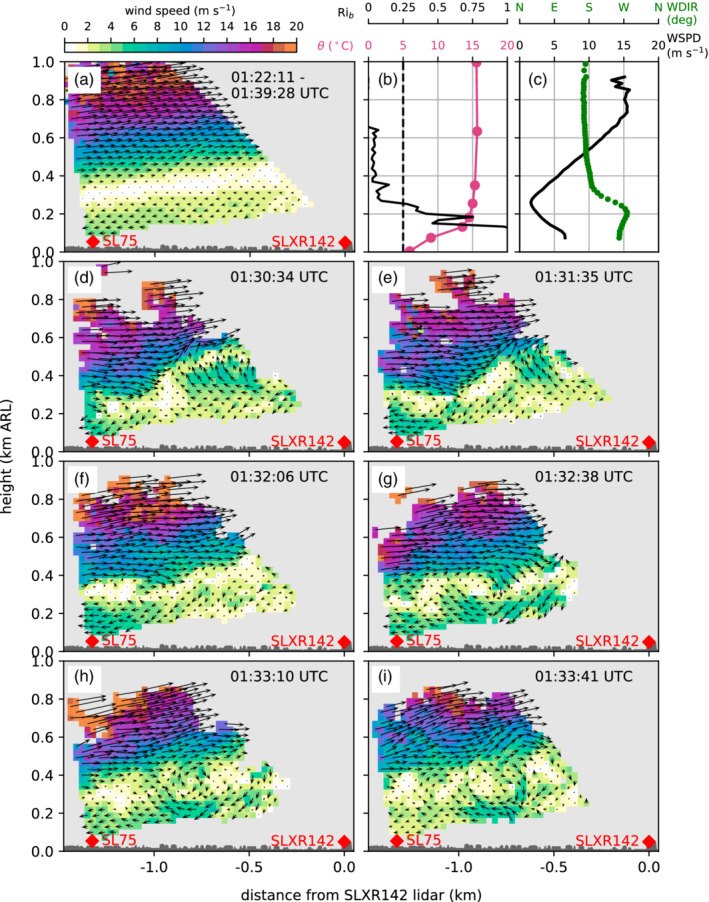
Two‐dimensional wind field on a southeast‐to‐northwest oriented vertical plane between the lidar SL75 and SLXR142 (RHIsn; Table 1 and Figure 2b) on 5 November 2017. (a) Mean wind field averaged between 0122 and 0140 UTC and (d)‐(i) wind field for six consecutive time steps between (d) 0130:34 UTC and (i) 0133:41 UTC. The time shown on each panel gives the starting time of the corresponding scan. The arrows represent the projection of the three‐dimensional wind vectors on the plane and the colours depict their magnitude. The height of the buildings along the transect is indicated in dark grey. (b) Pseudo‐vertical profile of potential temperature, θ, along the northern slope (SP_N; Figure 1b) and vertical profile of the bulk Richardson number, ${R\;i}_{b}$ (Section 2.2.3). The black dashed line marks ${R\;i}_{b}=0.25$. (c) Vertical profiles of horizontal wind speed, WSPD, and wind direction, WDIR, measured with the SL88 lidar. Profiles in (b) and (c) are averaged over the same period as in (a)

During the night from 4 to 5 November, the two‐dimensional wind field retrieved for the southeast‐to‐northwest orientated lidar plane (RHIsn; Table 1 and Figure 2b) reveal propagating roll‐up vortices that are evidence for KH instabilities. An example for six consecutive time steps between about 0130 and 0134 UTC on 5 November is shown in Figure 10d–i. The corresponding mean wind field averaged over 18 min highlights a pronounced shear flow with southerly foehn aloft and pre‐foehn westerlies underneath (Figure 10a). The pre‐foehn westerlies are represented as reversed flow towards the SL75 lidar in the lowest 200 m above the valley floor. Since the wind vectors are projected on the lidar plane, the magnitude of the pre‐foehn westerlies is lower than the horizontal winds retrieved from the SL88 measurements (Figure 10c). Above the pre‐foehn westerlies, a transition layer between about 200 and 600 m ARL is visible, which is characterized by a large spatial and temporal variability in the wind field (Figure 10d–i). On average, this layer is characterized by very low wind speeds (Figure 10a,c) and a near‐neutral stratification (Figure 10b). These atmospheric conditions result in a bulk Richardson number, ${R\;i}_{b}$, well below 0.25 in the transition layer above 200 m ARL (Figure 10b). Hence, the necessary condition for KH instability derived from linear theory, Ri<0.25 (Howard, 1961; Miles, 1961), is fulfilled. This instability causes the waves and eddies illustrated in Figure 10d–i. At the beginning of the shown sequence, a wave crest is located between the two lidars and extends from about 300 to 600 m ARL (Figure 10d,e). Within the period of four retrievals, this wave crest has propagated northward out of the detectable part of the vertical plane (Figure 10f). Soon afterwards, a new vortex forms with a diameter of about 300 m (Figure 10g) which propagates northward again (Figure 10h,i).

### 4.4 Final foehn breakthrough

In general, the foehn breakthrough marks the time when a station records foehn temperature. In this section, the *final breakthrough* of foehn denotes the phase on 5 November when ultimately all stations in the vicinity of Innsbruck observed foehn. An impression of the spatial variability of the breakthrough time can be gained from the time series of potential temperature (Figure 5a). The most easterly eddy‐covariance station EC_E reached foehn temperature first, followed by EC_C and EC_W.

**Figure 11:**
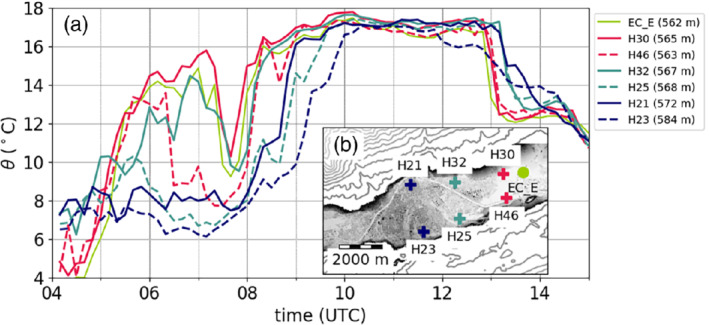
(a) Time series of potential temperature, θ, measured with T/RH loggers and the EC_E station during the period of the final foehn breakthrough on 5 November 2017. Same colours refer to loggers located approximately at the same longitude. Full (dashed) lines refer to T/RH loggers on the northern (southern) side of the Inn Valley. The locations of the stations are shown in (b) and their heights above m.s.l. appear in the legend

**Figure 12:**
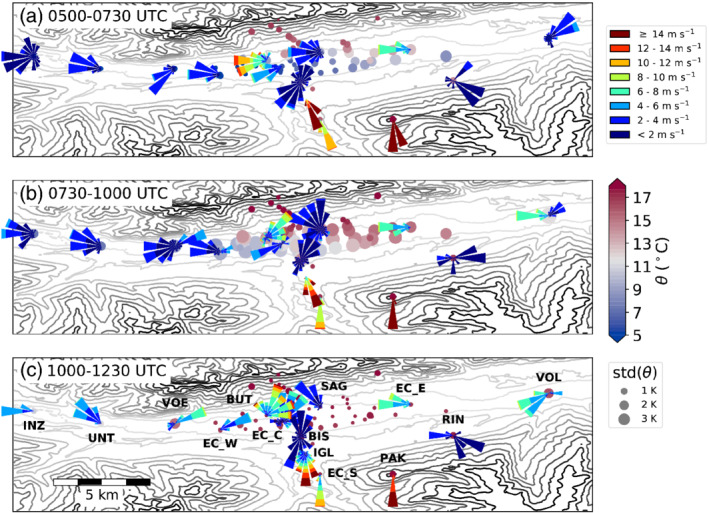
Map of potential temperature and wind measured at various surface stations for the three periods between (a) 0500 and 0730 UTC, (b) 0730 and 1000 UTC, and (c) 1000 and 1230 UTC on 5 November 2017. The colour and size of a station circle represents the mean and standard deviation of potential temperature over the specified period. Wind roses at selected stations represent the frequency distributions of wind speed and direction for the corresponding period. Labels of the stations with wind roses are given in (c). Terrain height is shown as contours with 200 m intervals. The three panels represent (a) the initial, transient foehn phase in the northeast of the city, (b) the phase of the final breakthrough with a westward propagation of the foehn–CAP air mass boundary, and (c) the fully developed foehn stage

Prior to the final breakthrough, the potential temperatures measured at EC_E already reached foehn temperature between about 0530 and 0720 UTC, followed by a short interruption (Figures 5a and 11). This initial transient foehn phase is also visible in the time series of other stations located to the east of Innsbruck (H30, H46, H32, EC_E in Figure 11). This phase was more pronounced and lasted longer at the northeastern stations (H30, H32, EC_E). The restricted spatial extent of this initial foehn phase is best visible in Figure 12a, which shows the potential temperature averaged between 0400 and 0730 UTC on 5 November at various stations and the corresponding standard deviation. In addition, the wind distribution for the same period is shown for selected stations. During these 2.5 hr, the stations close to the centre of Innsbruck and to the west record relatively low potential temperature (about 5 to 8 °C) and only small variability ($\leq 1^{\circ }$C). The wind measurements along the valley indicate pre‐foehn westerlies, which is in good agreement with the lidar‐retrieved horizontal wind speeds for this time (Figure 5c). At the same time, stations northeast of the city showed higher temperatures (about 10 to 14 °C) and higher variability ($\geq 2^{\circ }$C). Even though EC_E was affected by foehn during this period, its wind rose indicates predominantly westerly flow (Figure 12a). Nevertheless, the TKE at EC_E increased from below 1 m${}^{2}\cdot$s${}^{-2}$ to above 3 m${}^{2}\cdot$s${}^{-2}$, indicating enhanced mixing, while at the same time TKE decreased at EC_W and EC_C (Figure 5d). Consistent with this spatial difference in TKE, the temperature fluctuations in the CAP were larger along the northeastern slope profile SP_NE than along the northern profile SP_N closer to the city centre (Figure 6b). Furthermore, the averaged vertical wind speed in the foehn layer at the eastern lidar site SL74 changed from close to zero to about 1 m·s${}^{-1}$ (Figure 5e), indicating a change in the flow structure above the CAP. The averaged vertical wind speed in the foehn layer measured by the SL88 lidar behaved similarly to the SL74 lidar for the complete period of IOP2 (not shown).

The final breakthrough started shortly before 0800 UTC on 5 November. It was first detected at the stations which were influenced by the prior transient foehn phase (H30, EC_E, H32 and H46 in Figure 11). Depending on the location, the potential temperature increased by about 6 to 10 K at the time of the breakthrough. The dense network of T/RH loggers enabled observation of the horizontal propagation of the foehn–CAP air mass boundary across the city. The two stations H30 and EC_E, located at the valley floor furthest east of the city close to the northern slope, recorded the breakthrough first (Figure 11). About 20 min later, the two stations H32 and H46, located 2.3 km west and 1 km south of H30, respectively, observed the onset of foehn. Thus, the foehn–CAP boundary propagated southwestward and after another 40 to 50 min reached the stations H21 west and H25 south of H32, respectively. The spatial distribution of potential temperature averaged over time and its standard deviation between 0730 and 1000 UTC on 5 November (Figure 12b) supports the southwestward propagation. During the final breakthrough phase, all stations at the valley floor measured similar temporal variability in temperature and greater variability than in the previous period (cf. Figure 12a,b). However, stations in the northeast of the city observed higher mean potential temperature which resulted from the earlier breakthrough there (Figure 12b). During the breakthrough period, stations close to the city centre recorded predominantly northeasterly winds, while EC_E measured westerlies. Stations to the west were still under the influence of pre‐foehn westerlies. The weather station HIL located within the Doppler wind lidar triangle (Figure 1b) measured the breakthrough as a strong increase in potential temperature between 0850 and 0920 UTC (not shown). The horizontal wind fields derived from lidar data are rather chaotic with low wind speeds on average (not shown).

### 4.5 Flow splitting and rotor formation

After the final breakthrough between 1000 and 1230 UTC, the valley atmosphere above the city was well mixed. Hence, the temporal variability of potential temperature at the valley floor was low (Figure 12c). However, the foehn did not reach as far as the station UNT, 10 km west of Innsbruck. The two westernmost stations shown in Figure 12c remained in the CAP during the foehn event and recorded downvalley flow. In the foehn region, the stations east of the Wipp Valley exit (e.g., EC_E) observed westerly flow while stations west of the exit (e.g., EC_W) recorded easterly flow (Figure 12c). This change in wind direction along the Inn Valley over the city illustrates flow splitting and deflection into two foehn currents at the mountain range north of Innsbruck. Horizontal winds measured by the lidar SL88 were below 10 m·s${}^{-1}$ at all heights and hence lower than the southerly foehn flow above the CAP prior to the breakthrough (Figure 5c). Stations aligned in the extension of the Wipp Valley exit measured weak winds from various directions, while stations to the north measured mostly wind from north (Figure 12c).

**Figure 13:**
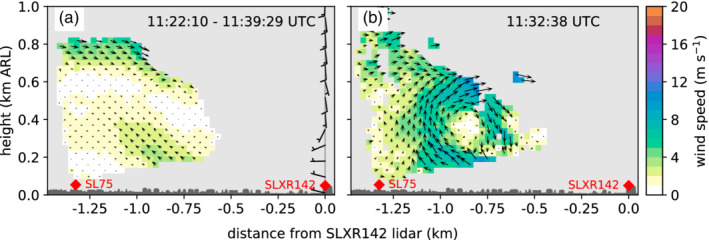
Two‐dimensional wind fields on a southeast‐to‐northwest oriented vertical plane between the lidar SL75 and SLXR142 (RHIsn; Table 1 and Figure 2b). (a) Averaged wind field between 1122 and 1140 UTC on 5 November (32 scans). Wind barbs above the SLXR142 lidar location show the vertical profile of horizontal wind measured with SL88 lidar for the same period (also Figure 5c). (b) Two‐dimensional wind field retrieved from a single dual‐Doppler scan included in the averaging period of (a)

**Figure 14:**
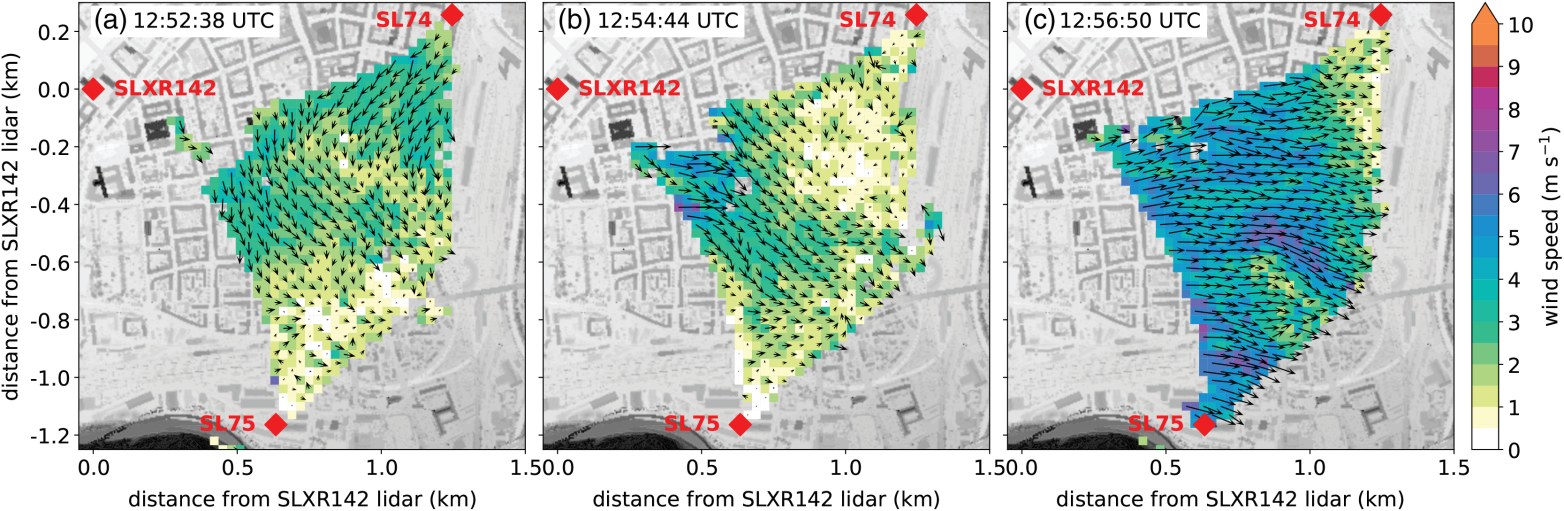
Spatial distribution of the horizontal wind at (a) 1252:38 UTC, (b) 1254:44 UTC and (c) 1256:50 UTC on 5 November 2017 retrieved from coplanar scans performed with three multiple Doppler wind lidars (PPI3; Table 1 and Figure 2c). Colours represent the magnitudes of the horizontal wind vectors shown as arrows. The locations of the lidars are shown as red diamonds. The building height is shown as grey shading

Between 1040 and 1200 UTC on 5 November, the foehn flow in the lowest 800 m above the SL88 lidar changed direction from south to north (Figure 5c). This reversed foehn flow from the north indicates the formation of an atmospheric rotor above the city centre. The valley atmosphere during this event was completely mixed (Figure 5a,b). In the two‐dimensional wind field on the southeast‐to‐northwest oriented vertical plane averaged between 1122 and 1140 UTC on 5 November, the rotor is seen as a weak northerly wind below 600 m ARL and a moderate southerly flow above 800 m ARL (Figure 13a). This wind reversal is also seen in the vertical profile of the horizontal wind measured with the SL88 lidar and averaged over 18 min (wind barbs in Figure 13a). Instantaneous two‐dimensional wind fields from this period (e.g., Figure 13b) show the presence of smaller‐scale eddies, called subrotors in earlier studies (e.g., Doyle *et al*., 2009; Hill *et al*., 2010). In contrast to the KH instabilities at the CAP–foehn interface shown before (Figure 10), the subrotors occurred in a well‐mixed environment with only weak mean shear on the lidar plane (Figure 13a). Horizontal wind fields retrieved from lidar data (PPI3; Table 1 and Figure 2c) show northerly winds on the whole lidar plane during this period. One example for this reversed flow at a later time (1253 UTC) is shown in Figure 14a. Evidently, the footprint of the rotor represented by the low‐level reversed flow was larger than 1 km${}^{2}$. At SL74 and SL88 instantaneous downdraughts of up to 8 m·s${}^{-1}$ were detected, resulting in vertical velocity variances of more than 6 m${}^{2}\cdot$s${}^{-2}$ (not shown), while mean downdraughts averaged over 30 min had a magnitude of less than 2 m·s${}^{-1}$ (Figure 5e).

### 4.6 Foehn breakdown

Stations located at the bottom of the Inn Valley and slightly above observed the foehn breakdown as a sudden drop in potential temperature at around 1300 UTC on 5 November 2017 (e.g., Figure 5a). This initial sudden cooling occurred in a deeper layer east and west of the city (up to about 150 m) than at the city centre, as documented by the slope profiles (not shown).

**Figure 15:**
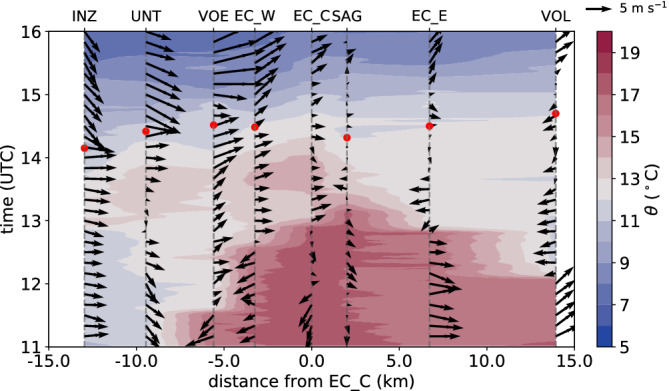
Temporal evolution of potential temperature (θ, colour shading, interval 1 K) and horizontal wind (vectors) near the surface along the Inn Valley from west to east based on station measurements (Figure 1a) during the breakdown of foehn on 5 November 2017. The starting time of measured precipitation at each location is marked by red dots

The foehn breakdown was first observed west and east of the city, before it was detected in the city centre. In VOE (5 km west of the centre) the breakdown occurred at about 1130 UTC and in VOL (13 km east) at about 1205 UTC, while foehn still prevailed at stations in between at this time (Figure 15). For most stations, the drop in potential temperature was accompanied by a change in wind direction. Stations east of the city centre (SAG, EC_E and VOL) observed a shift from westerly to easterly flow and vice versa at stations west of the city (VOE, EC_W; Figure 15). Hence, the breakdown of foehn manifested itself as colder air flowing from east and west towards the city centre and lifting the foehn flow from the valley floor. There is evidence that the CAP pushed back from both sides prior to the arrival of the cold front. For example, the most western station INZ remained in the CAP during the whole foehn period (Figures 12a–c and 15). It recorded the first cooling associated with the arriving cold front at about 1400 UTC, which is about 1 hr after the first cooling at the more eastern stations VOL and EC_E was observed (Figure 15). The arrival of the cold front at INZ was characterized by a wind shift from west to northwest and the onset of precipitation. INZ lies southeast of a mountain pass (Seefeld Saddle) which is a preferred entrance into the Inn Valley for cold fronts approaching from the north (e.g., Gohm *et al*., 2010). The backflow of the CAP occurred faster from the east (∼2.2 m·s${}^{-1}$) than from the west (∼0.6 m·s${}^{-1}$; Figure 15). The sudden backflow of the CAP over the city centre was captured with coplanar PPI scans between about 1253 and 1257 UTC. Three consecutive horizontal wind fields illustrate the development during the CAP backflow (Figure 14). The first field is characterized by predominantly northerly (reversed) foehn flow below 3 m·s${}^{-1}$, which represents the previously mentioned rotor (Figure 14a). Two minutes later, moderate westerlies start to be visible in the northwestern part of the lidar triangle (Figure 14b). This coincides with a wind shift and an associated cooling at EC_C (Figure 15), located next to the SLXR142 lidar. Another 2 min later, the CAP backflow covered the whole lidar plane (Figure 14c) and terminated the foehn in the city centre at the surface. Aloft weak foehn flow prevailed for about another hour (PAK in Figure 3a).

Ultimately, the foehn event was terminated by the arrival of a cold front. As mentioned above, the most westerly station (INZ) recorded the cold front passage at about 1400 UTC, illustrated by cooling and the start of precipitation (Figure 15). The cold front propagated eastwards (downvalley) which resulted in cooling and precipitation first in the west and later in the east of the city (Figure 15). Only at SAG precipitation was already observed earlier. This station was probably affected by precipitation spill‐over due to its close location to the northern mountain range (Nordkette; Figure 1b). In the Wipp Valley at ELL, the cold front arrived at 1440 UTC and at the mountain station PAK at 1500 UTC, as illustrated by a shift from southerly to northerly flow (Figure 3c).

## 5 DISCUSSION

In this section, the phases of foehn–CAP interaction presented above are discussed with regard to different processes and compared to the literature. Their influence on the structure and temporal evolution of the CAP is evaluated and their contribution to the final foehn breakthrough analysed. Furthermore, a conceptual model is presented for the foehn–CAP interaction during the two IOP nights, the foehn breakthrough and breakdown.

### 5.1 Shear‐induced gravity waves

During the first half of the night from 3 to 4 November 2017, nearly periodic up‐ and downdraughts were observed for a 6 hr period at all lidar sites (Section 4.1). These oscillations were first visible at the top of the CAP and extended down to the valley bottom during the night. Together with their growth in vertical extent, vertical velocities increased from 1 to 6 m·s${}^{-1}$. The oscillations coexisted with a shear layer between the CAP and the foehn flow. In this transition zone, smaller fluctuations were visible and most likely related to shear flow instabilities (Figure 7d). For a stably stratified flow, Davis and Peltier (1979) showed that beside KH disturbances resonant modes can be excited by shear which have longer wavelengths and periods. These resonant modes are characterized by small growing rates and are relatively unimportant in terms of their impact on the initial state. Nater (1979) analysed shear‐induced gravity waves which formed at the foehn–CAP interface. He applied Wegener's hypothesis (Wegener, 1906) which describes the propagation of gravity waves along the interface between two layers with different densities and different flow velocities $v_{l}$ (lower layer) and $v_{u}$ (upper layer). For such a two‐layer system, the mean flow vector is defined as $v_{m}=0.5(v_{l}+v_{u})$ and the shear vector is $v_{s}=v_{u}-v_{l}$. The phase speed, $c_{p}$, of the waves is given by the magnitude of the mean flow vector, i.e., $c_{p}=|v_{m}|$, whereas the direction of the wave propagation is given by the shear vector $v_{s}$. In our case, $v_{l}$ represents the vertically averaged wind in the CAP, and $v_{u}$ the mean foehn flow aloft. The wind vector in the CAP is taken from the lidar‐retrieved horizontal wind measurements. Due to a limited range of the SL88 lidar, the wind vector in the foehn flow is taken from the mountain station PAK.

For the period between 2000 UTC on 3 November and 0100 UTC on 4 November, mean wind vectors are $v_{l}=(4.9,0.2)$ m·s${}^{-1}$ and $v_{u}=(-2.2,14.5)$ m·s${}^{-1}$. Hence, the shear vector is $v_{s}=(-7.1,14.3)$ m·s${}^{-1}$ which points in the direction of 334° from north. This is in good agreement with the observed propagation direction of 342° (Section 4.1). The mean wind speed of the two layers is $|v_{m}|=7.5$ m·s${}^{-1}$, which is close to the observed propagation speed of 6.3 m·s${}^{-1}$. Multiplying the previously estimated oscillation period of the vertical velocity variance, τ=255 s (Section 4.1), by the theoretical phase speed of 7.5 m·s${}^{-1}$, a wavelength of ∼1,900 m is deduced. This is larger than the observed wavelengths of 1,600 and 1,300 m. One reason for this discrepancy is the usage of the mountain station PAK for the wind vector in the foehn flow, $v_{u}$. It is likely that winds at PAK (1,680 m ARL) were stronger than the winds interacting with the CAP.

The patterns of observed horizontal flow divergence illustrate that these oscillations also affected the flow field at the valley floor (Figure 8). However, they did not have an impact on the measured temperatures there; neither distinct temperature oscillations nor an indication for mixing with warmer air could be detected. This is in agreement with the work of Davis and Peltier (1979). Only the sensors at 350 and 634 m ARL measured an increase in temperature between 1900 UTC on 4 November and 0100 UTC on 5 November (T2 and T3 in Figure 5b). These sensors were located at the height of the CAP–foehn interaction zone, where smaller frequencies and higher vertical velocity variances occurred (Figure 7d–f).

### 5.2 Shear flow instabilities and turbulent mixing

During both IOP nights, observations suggest the presence of shear flow instabilities; from 3 to 4 November 2017, fluctuations in the order of 1 to 2 min were present in the interaction zone between pre‐foehn westerlies and the southerly foehn flow above (Figure 7d,e). This layer was characterized by vertical velocity variances of more than 3 m${}^{2}\cdot$s${}^{-2}$. In the second IOP night from 4 to 5 November, KH instabilities were identified at the interface of the CAP and the foehn flow (Figure 10a–f). Thus, there is evidence that for both IOP nights turbulent mixing was present between the foehn flow and the underlying CAP. In the first IOP night, the depth of the CAP decreased between 1800 UTC on 3 November and about 0300 UTC on 4 November (Figure 5b). Potential temperatures near the Inn Valley bottom remained nearly constant or increased slightly from 0000 UTC on 4 November onwards (e.g., EC_W in Figure 5a). During the second night, the top of the CAP stayed at a constant level of about 200 m ARL (Figure 5b), however the lowest level of the foehn flow descended during the course of the night (Figure 5b,c). For both nights, the question arises to what extent the turbulent mixing contributed to the CAP's removal. In order to answer this question, the heating/cooling rate due to turbulent mixing has to be quantified and assessed relative to other terms in the heat budget. However, based on our measurements, we cannot determine all terms explicitly and are therefore not able to fully close the budget. Neglecting moist processes, radiative flux divergence, and turbulent mixing in the horizontal, the heat budget is given by (e.g., Wyngaard, 2010, equation 8.67):

(7) $$\underset{\text{NET}}{\underbrace{\frac{\partial \bar{\theta }}{\partial t}}}=\underset{\text{ADV}}{\underbrace{-\left(\bar{u}\frac{\partial \bar{\theta }}{\partial x}+\bar{v}\frac{\partial \bar{\theta }}{\partial y}+\bar{w}\frac{\partial \bar{\theta }}{\partial z}\right)}}\underset{{\text{TRB}}_{z}}{\underbrace{-\frac{\partial \bar{\theta^{{}^{\prime }}w^{\prime }}}{\partial z}}}.$$

Here, overbar and prime denote ensemble mean and turbulent fluctuation, respectively. According to Equation (7), the net potential temperature tendency (NET) is given by the sum of the three components of temperature advection (ADV) and the vertical turbulent heat flux divergence (TRB${}_{z}$). With the available observations, the turbulent heat flux, $\bar{w^{\prime }\theta^{\prime }}$, is estimated using K‐theory:

(8) $$\bar{w^{\prime }\theta^{\prime }}=-K_{h}\frac{\partial \bar{\theta }}{\partial z}\approx -K_{h}\frac{\Delta \bar{\theta }}{\Delta z}.$$

Here, $K_{h}$ is the eddy diffusivity for heat which is related to the eddy viscosity, $K_{m}$, through the turbulent Prandtl number, $Pr_{t}$:

(9) $$K_{h}=\frac{1}{{\mathrm{Pr}}_{t}}K_{m}.$$

Following the parametrization approach of Deardorff (1980), $K_{m}$ is a function of the turbulence kinetic energy, TKE, and the mixing length ℓ:

(10) $$K_{m}=0.1\ell \sqrt{\text{TKE}}.$$

For stable stratification, ℓ is determined by

(11) $$\ell =0.76\sqrt{\text{TKE}}{\left(\frac{g}{\bar{\theta_{0}}}\frac{\Delta \bar{\theta }}{\Delta z}\right)}^{-1/2},$$

where $\bar{\theta_{0}}$ is the mean potential temperature of the considered layer. In Deardorff (1980), the turbulent Prandtl number depends on the model grid size (and mixing lengths larger than the model scale are not allowed), since this parametrization was developed to represent the subgrid‐scale turbulence. However, here we assume that TKE and ℓ are representative for the complete spectrum of turbulence, or at least the dominant part. For simplicity, we chose $Pr_{t}=1$ since for both nights the static stability is either stable or slightly neutral (Figure 6) (e.g., figure 9 of Webb, 1970). Assuming isotropic turbulence, TKE is deduced from the vertical velocity variance measured with the SL74 lidar (Section 2.2.1) by:

(12) $$\text{TKE}=\frac{1}{2}\left(\bar{u^{\prime 2}}+\bar{v^{\prime 2}}+\bar{w^{\prime 2}}\right)\approx \frac{3}{2}\bar{w^{\prime 2}}.$$

The mean vertical temperature gradient is calculated from observations along the slope profile SP_N.

**Figure 16:**
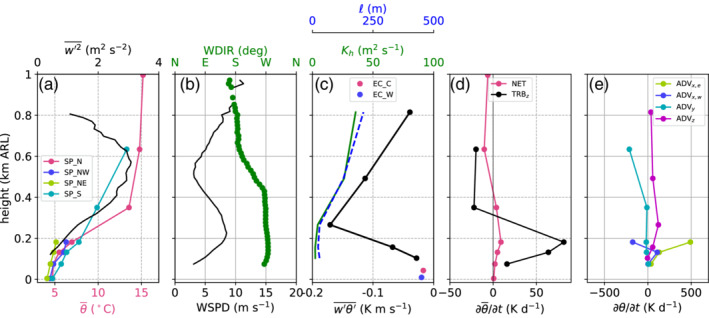
(a) Vertical profile of vertical velocity variance measured with the SL74 lidar (black line) and profiles of mean potential temperature along SP_N, SP_NW, SP_NE and SP_S (see legend) averaged between 0000 and 0400 UTC on 4 November 2017. (b) Vertical profiles of horizontal wind speed (black line) and wind direction (green line) measured with SL88 lidar and averaged for the same period. (c) Parametrized mixing length, ℓ, eddy diffusivity of heat, $K_{h}$, and vertical turbulent heat flux, $\bar{w^{\prime }\theta^{\prime }}$, derived for the same period from Equation (8), for different layers. Additionally the averaged vertical turbulent heat fluxes measured at the eddy‐covariance stations EC_C and EC_W are shown. (d) Net tendency of potential temperature, NET, calculated for the same period from measurements along SP_N and temperature tendency resulting from turbulent vertical heat flux divergence, TRB${}_{z}$, calculated from the profile shown in (c). (e) Tendencies of potential temperature representing horizontal (ADV${}_{x,w}$, ADV${}_{x,e}$ and ADV${}_{y}$) and vertical (ADV${}_{z}$) advection. Difference between ADV${}_{x,w}$ and ADV${}_{x,e}$ are explained in the main text. See also Equation (7)

Between 0000 and 0400 UTC on 4 November, the averaged potential temperature profile indicates highest stability in the layer 200 to 400 m ARL between the CAP and the foehn flow (Figure 16a, pink line). The variance of vertical velocity reaches its highest values at around 600 m ARL (Figure 16a, black line). This leads to a peak in the downward turbulent heat flux of about −0.17 K·m·s${}^{-1}$ at 300 m ARL (Figure 16c, black line), where the atmosphere is still stably stratified and turbulent motions are strong. Above (below) this level, vertical heat flux divergence (convergence) occurs. The estimated heat flux at the lowest level (about 100 m ARL) has a comparable magnitude to vertical turbulent heat flux measured at the eddy‐covariance station EC_C (43 m AGL) and EC_W (2.5 m AGL), respectively (pink and blue markers in Figure 16c). Form the calculated vertical heat fluxes, the associated heating rate can be deduced with

(13) $${\left(\frac{\partial \bar{\theta }}{\partial t}\right)}_{{\text{TRB}}_{z}}\approx -\frac{\Delta \bar{w^{\prime }\theta^{\prime }}}{\Delta z}.$$

This leads to heating rates due to turbulent mixing of up to 80 K·d${}^{-1}$ in the CAP and cooling rates of about −20 K·d${}^{-1}$ in the foehn flow (Figure 16d, black line). This vertical structure is consistent with the observed net tendency (Figure 16d, pink line) which represents the linear trend of temperature time series over the averaging period of 4 hr. However, the heating rate due to vertical mixing in the CAP is more than ten times larger than the observed net heating rate. This indicates that heating caused by vertical turbulent mixing is partly compensated by cooling from other heat budget terms (Equation (7)).

**Figure 17:**
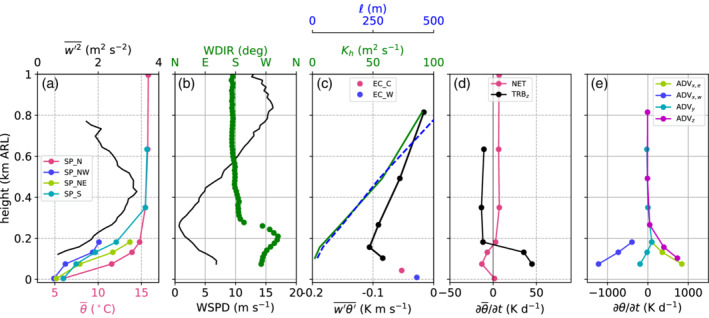
As Figure 16, but for the period between 0000 and 0400 UTC on 5 November 2017. The x‐axis range in (e) differs from that shown in Figure 16e

Between 0000 and 0400 UTC on 5 November, the CAP is shallower and more stable than during the night before and the foehn flow less stable (Figure 17a, pink line). The vertical velocity variance has similar magnitudes to the first IOP night, with a peak located about 200 m lower (Figure 17a, black line). This situation leads to smaller turbulent heat fluxes (of about −0.1 K·m·s${}^{-1}$ below 200 m ARL; Figure 17c, black line) than the night before, but a similar vertical structure of turbulent heating with warming in the CAP and cooling in the foehn flow (Figure 17d, black line). However, for the second night, this warming by turbulent mixing in the CAP cannot prevent a net cooling (Figure 17d, pink line). Similarly, the foehn flow experienced net warming despite cooling due to vertical turbulent heat flux divergence (Figure 17). This analysis shows that, for both nights, the other terms of the heat budget (Equation (7)) such as temperature advection and horizontal heat flux convergence, cannot be neglected.

In the CAP, horizontal temperature advection is non‐zero in case of pre‐foehn westerlies combined with horizontal temperature gradients resulting from CAP heterogeneity. In the first night (Figure 16a), the mean potential temperature profiles west and east of the city (SP_NW and SP_NE) were nearly identical to the profile near the city centre (SP_N) and the pre‐foehn westerlies formed a uniform layer of around 400 m depth (Figure 16a,b). Using the slope profile of potential temperature and the horizontal wind measured with the SL88 lidar, horizontal advection terms and the associated heating can be estimated. The advection component in the x‐direction (ADV${}_{x}$) estimated in two different ways by calculating the horizontal gradient between SP_NW and SP_N (ADV${}_{x,w}$) and between SP_N and SP_NE (ADV${}_{x,e}$). Further, the vertical advection can be derived from the potential temperatures measured along SP_N and the vertical profiles of mean vertical velocities derived from SL74. The resulting heating rates for the three components of the advection term and their sum are given in Figure 16e for the analysed period. It can be seen that the advection terms have much larger magnitudes than the vertical heat flux divergence. Considering the extremely rough estimation of these terms, it is conceivable that, for the shown case, the advection processes compensated each other and turbulent erosion was of major importance, similar to the findings of Lareau and Horel (2015b). During the second night (Figure 17a), the profiles of potential temperature in the west (SP_NW), east (SP_NE) and in the south (SP_S) were substantially colder than near the city centre (SP_N), resulting in much larger temperature tendencies due to horizontal advection (Figure 17e). The pre‐foehn westerlies in combination with the CAP heterogeneity led to negative horizontal advection from the west (ADV${}_{x,w}$) and south (ADV${}_{y}$) which is partly compensated by warming due to vertical advection (ADV${}_{z}$). Hence, advection acted against vertical turbulent heat flux convergence – presumably even overcompensating the effect of turbulent mixing. However, the advection term estimated between SP_NE and SP_N (ADV${}_{x,e}$) resulted in warm air advection towards the east of Innsbruck, This is a result of the CAP deformation with a minimum in CAP depth in the vicinity of the city centre. This discrepancy between ADV${}_{x,w}$ and ADV${}_{x,e}$ shows that our approach is sensitive to the location of the temperature measurements in the CAP. Further, the horizontal distance between the two temperature profiles used to estimate advection is about 2.5 km. It could well be that mean CAP heterogeneity is characterized by smaller‐scale temperature gradients not captured by our observations. This would imply that the estimated horizontal temperature advection is not representative of the heat budget near the city centre. With our limited dataset we cannot test this hypothesis. Hence, for a complete and consistent heat budget analysis, large‐eddy simulations are needed.

The quality of our turbulent heat flux estimates is determined by the applicability of the Deardorff (1980) parametrization and the isotropy assumption. For KH instability at the top of a CAP, Gubser and Richner (2001) estimated turbulent heat fluxes of –15 W·m${}^{-2}$ and Lareau and Horel (2015a) simulated values of −100 to −150 W·m${}^{-2}$. Hence, our estimated heat fluxes in the range of −100 to −170 W·m${}^{-2}$ (−0.1 to −0.17 K·m·s${}^{-1}$; Figures 16a and 17a) are in the same range as the simulated ones. Nevertheless, it is likely that we underestimate the true heat flux due to the assumption of isotropic turbulence. For example, Jiang and Doyle (2004) have shown that in breaking mountain waves the horizontal contribution to TKE can be larger than the vertical contribution. Applied to our case, this would mean that, with TKE solely based on $\bar{{w{}^{\prime }}^{2}}$, we would underestimate TKE and therefore also $\bar{w^{\prime }\theta^{\prime }}$.

**Figure 18:**
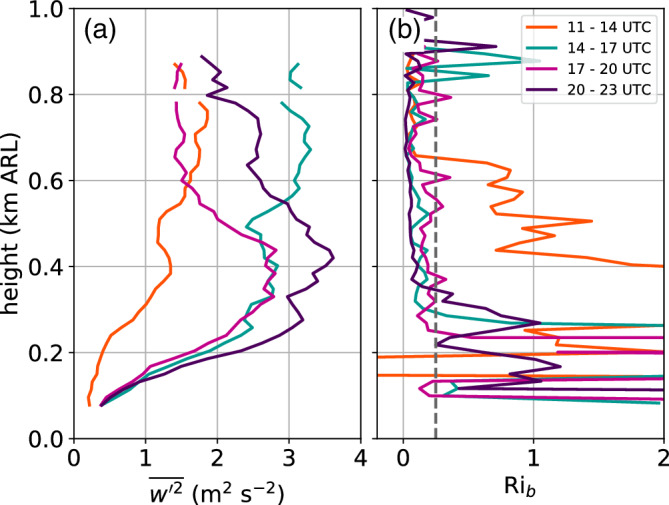
Three‐hourly averaged profiles of (a) vertical velocity variance, $\bar{w^{\prime 2}}$, and (b) bulk Richardson number, ${R\;i}_{b}$, for four different periods on 4 November 2017 (see legend). Vertical velocity variance is determined from data of the SL88 lidar. Bulk Richardson number is calculated from the vertical profiles of horizontal winds retrieved from SL88 measurements and the profiles of potential temperature along the SP_N slope profile (Section 2.2.3). The critical value of ${R\;i}_{b}=0.25$ is indicated by the dashed grey line

The transient breakthrough between 1400 and 1930 UTC on 4 November was characterized by fluctuations in the potential temperature (Section 4.2). Shear flow instability is indicated by the evolution of the vertical velocity variances measured above the city. Figure 18a shows profiles of vertical velocity variance averaged over three‐hourly periods. Between 1100 and 1400 UTC, variances stay below 2 m${}^{2}\cdot$s${}^{-2}$ for the whole profile. They increased to about 3 m${}^{2}\cdot$s${}^{-2}$ during the partial foehn breakthrough (1400–1700 UTC) and stayed at this higher level for the two subsequent periods. This suggest that turbulence and therefore mixing was increased during these periods. For the evolution of shear flow instabilities, the Richardson number has to fall below its critical value of 0.25 (e.g., Petkovšek, 1992). Vertical profiles of the bulk Richardson numbers, $i_{b}$, are determined for the four three‐hourly periods using the profiles of horizontal winds from the SL88 lidar and the profiles of the potential temperature measured along the northern slope SP_N (Section 2.2.3). For the period between 1100 and 1400 UTC, the Richardson number was subcritical above 650 m ARL (Figure 18b). In the three following periods, the layer of subcritical ${R\;i}_{b}$ extended further downward to 200 to 400 m ARL. Hence, shear flow instability was likely to occur in a deeper layer and closer to the surface. After the period of the transient foehn breakthrough (2000–2300 UTC), still rather high velocity variances were observed together with subcritical ${R\;i}_{b}$ above 400 m. However, associated shear‐induced mixing was not able to prevent the reformation of a night‐time CAP. Fluctuations in potential temperature were measured at the slope station BUT, 152 m above the city (Figure 9a), indicating that shear flow instabilities still occurred but were not able to penetrate deeper into the CAP. The lower depth of the CAP in the profile SP_N, i.e., further downstream of the Wipp Valley exit than the profiles further west (SP_NW) and east (SP_NE) (cf. Section 3.2 and Figure 6b), could be another indication for shear flow instabilities being more vigorous below the foehn jet emanating from the Wipp Valley. However, the lower CAP depth in the slope profile could also be the result of downward flow deflection at the slope of the Nordkette.

It is also possible that the observed turbulence on 4 November and the subcritical Richardson number are related to breaking gravity waves above the CAP. However, similar patterns of KH instability as illustrated in Figure 10 for 5 November were also observed in the afternoon of 4 November (not shown).

### 5.3 Flow deflection

Transient breakthrough of foehn was observed in two phases of the IOP: in the afternoon of 4 November and prior to the breakthrough on 5 November 2017. Both occurred first on the northern side of the Inn Valley and were accompanied by northerly winds (Figures 9b and 12a). This suggests that the transient intrusion of foehn air to the valley floor originated from foehn branches deflected at Nordkette. Nevertheless the two events differ from each other in terms of breakthrough time, location and development.

The first transient breakthrough (4 November) was observed during daytime. Thus, prior to the breakthrough, the CAP in the Inn Valley was destabilized by the surface sensible heat flux resulting from solar radiation (Figure 5b). However, the potential temperatures in the Inn Valley stayed below foehn temperature until the afternoon (Figure 9a). After about 1400 UTC, intermittent intrusion of foehn air, characterized by fluctuations of potential temperature, occurred first in the northern part, and later in the central and western part of the city, but never in the eastern part. These fluctuations suggest that smaller branches of foehn intruded into the CAP without widespread CAP displacement. One potential reason could be the shear flow instabilities that were present during this period (Section 5.2). Associated eddies on the top of the CAP may have grown across the city and may even have been deflected at Nordkette as they impinged on this mountain range. Both effects would have enhanced mixing in the northern part of the city, somewhat similar to the case 2 discussed by Fritts *et al*. (2010) (their figure 8).

The second transient breakthrough (5 November) occurred as a single abrupt temperature increase without fluctuations and only in the eastern part of the city in the morning before sunrise (≈0500 UTC; Figure 11). Hence, a potential contribution of solar radiation to this transient breakthrough can be excluded. We believe that this breakthrough was caused by an eastward‐deflected foehn branch displacing the CAP locally. Already in previous studies, the earlier breakthrough in the east of the city has been explained by flow splitting at Nordkette and a preferential eastward deflection of the foehn flow (e.g., Zängl and Gohm, 2006). There it was argued that the foehn flow exiting the Wipp Valley impinges with an oblique angle on the Nordkette, favouring a stronger eastward deflection. The short foehn interruption from about 0730 UTC until the final breakthrough in the east (Figure 11) was most likely caused by the CAP pushing back and lifting the foehn flow from the valley floor.

At the beginning of both transient breakthrough phases, a sudden increase in the mean vertical velocity of the foehn flow from initial values close to zero illustrates the formation of sustained updraughts (Figure 5e). On 3 November, these updraughts were observed close to the Wipp Valley's exit (SL75) and on 4 November also above the city centre (SL74). It is likely that these updraughts represent the ascending part of a gravity wave or a hydraulic jump. The latter has already been detected in the Inn Valley downstream of Patscherkofel in previous studies based on aircraft observations and simulations (e.g., Gohm and Mayr, 2004; Gohm *et al*., 2004; Weissmann *et al*., 2004). The formation, position and amplitude of gravity waves and associated hydraulic jumps have shown to be controlled thermally (e.g., Jiang and Doyle, 2008) or dynamically (e.g., Sheridan and Vosper, 2012; Elvidge *et al*., 2016; Strauss *et al*., 2016). Given the increasing cross‐Alpine pressure gradient (Figure 5d), and the changing vertical structure of the foehn in the Wipp Valley (Figure 4) during IOP2, a dynamical factor influencing the position of the observed updraughts is likely.

### 5.4 Conceptual model

**Figure 19:**
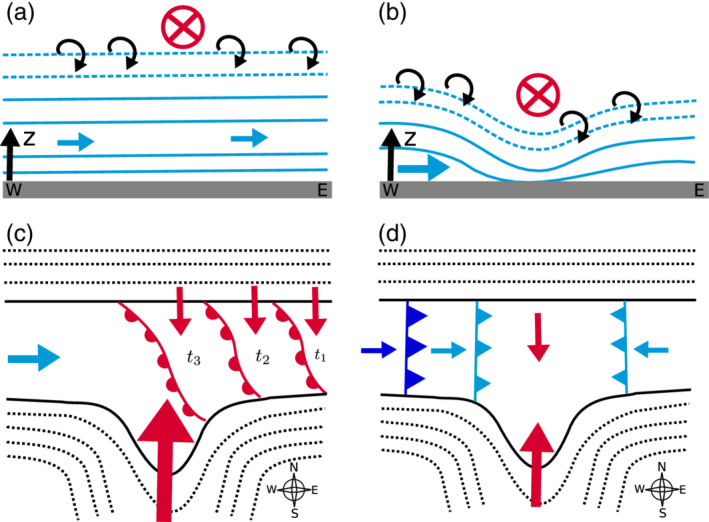
Schematic diagrams of turbulent erosion of the CAP in the Inn Valley during (a) the first and (b) the second night of IOP2, (c) the foehn breakthrough and (d) the foehn breakdown. (a, b) Vertical cross‐sections along the valley with blue lines indicating the isentropes inside the CAP and dashed blue lines representing isentropes in the CAP–foehn interaction zone. Blue arrows represent the pre‐foehn westerlies and the red arrow into the plane the upper‐level foehn flow. Curly black arrows indicate the presence of turbulence. (c, d) Top views of the Wipp Valley exit and the Inn Valley in the vicinity of Innsbruck. Dashed black lines indicate terrain contours and solid black lines highlight the floor of the Inn Valley. (c) The westward propagating foehn–CAP boundary is illustrated by a warm front at three different times ($t_{1}$, $t_{2}$ and $t_{3}$) accompanied by foehn air deflected at the mountain ridge north of Innsbruck (red arrows pointing southwards). (d) In the Inn Valley, a reversed foehn flow due to a rotor is indicated by a southward pointing red arrow. Foehn breakdown resulted from a CAP backflow from both sides (light blue cold fronts) prior to the arrival of the actual cold front (dark blue cold front). The size of the wind vectors represents the strength of the respective flows

The results shown in Section 4 and the estimation of the turbulent mixing in Section 5.2 are condensed into four schematic diagrams (Figure 19). Figure 19a,b summarize the situation in the Inn Valley during the first and second nights of IOP2, respectively. In the first night from 3 to 4 November, the CAP was relatively thick with a nearly homogeneous depth along the valley (Figure 19a). The estimated heating caused by vertical turbulent heat flux divergence is partly compensated by other terms in the heat budget. However, cooling terms are not efficient enough to prevent a net heating of the CAP. During the second night from 4 to 5 November, the CAP was much shallower and showed a minimum in depth downstream of the Wipp Valley exit (Figure 19b). This heterogeneity in the CAP thickness resulted in horizontal pressure gradients and compensation flows which caused advection of cold air towards the region of the CAP minimum and warm air advection downvalley. This cold air advection most likely overcompensated turbulent CAP erosion.

The foehn breakthrough on 5 November 2017 (Figure 19c) was first observed east and downstream of the Wipp Valley exit (cf., Figure 11) and was accompanied by foehn air deflected at Nordkette. In the course of the breakthrough, the foehn–CAP boundary propagated westwards until all stations in the vicinity of Innsbruck recorded foehn. After the final breakthrough in the Inn Valley, a rotor formed above the city (Figure 13), indicated as near‐surface reversed flow in Figure 19d. This event shows similarities to a rotor scenario described by Strauss *et al*. (2016) (their figure 16d). There, a large rotor formed between two ridges, completely controlling the valley flow. However, this rotor formation was accompanied by strong cross‐mountain winds, while in our case the wind speed measured at PAK (Figure 3b) was weaker than in earlier periods.

The foehn air in the Inn Valley was finally displaced by the CAP pushing back from both valley sides (Figure 19d). Later, the event was finally terminated by a cold front arriving from the west. Previous studies of the foehn breakdown in the area of Innsbruck identified the passage of a cold front as the main reason (e.g., Gohm *et al*., 2010). However, for this case, a CAP backflow was found to be the first process. This was only possible since the CAP's removal was spatially restricted to the area around Innsbruck and the CAP remained to the east and west. Similarly, Lareau and Horel (2015a) found for the Salt Lake Basin a lateral displacement of the CAP due to a gravity wave, with a recovery of the CAP afterwards.

## 6 CONCLUSION

A case‐study of the fine‐scale structure of an Alpine south foehn event over the city of Innsbruck (Austria) is presented. The foehn flow emanated from the south–north aligned Wipp Valley and encountered a cold‐air pool (CAP) in the west–east orientated Inn Valley. The goal was to identify dominant processes of foehn–CAP interaction and to evaluate their contribution to the CAP dissipation. The event occurred between 3 and 5 November 2017 during the second Intensive Observation Period (IOP2) of the Penetration and Interruption of Alpine Foehn (PIANO) field campaign. For the analysis we used data from automatic weather and eddy covariance stations located in both valleys, radiosonde ascents, a network of temperature and humidity loggers and four Doppler wind lidars. From single lidar measurements, vertical profiles of the mean horizontal wind, the mean vertical velocity and the vertical velocity variance were deduced. By performing coplanar scans, the two‐dimensional wind field on horizontal and vertical planes could be determined.

The event started on 3 November as a shallow foehn, which developed into a deep foehn on 4 November and was terminated on 5 November by the passage of a cold front. While foehn persisted in the upper Wipp Valley during the entire IOP, a CAP formed in the Inn Valley during both nights and prevented foehn reaching the valley floor during night‐time. This CAP was thicker, spatially more homogeneous and less stable in the first night. In the second night, the CAP was characterized by large spatial variability in depth and stability. In the afternoon of 4 November and in the morning of 5 November, the foehn penetrated to the bottom of the Inn Valley. On the first day, foehn breakthrough was transient and occurred only in the central and western part of the city of Innsbruck. Hence, the CAP was not completely eroded. In contrast, on the second day, the breakthrough occurred first in the northeastern part of the city. Subsequently the foehn–CAP boundary progressed westward across the city resulting in a complete CAP removal in the greater city area. Different phases of foehn‐CAP interactions could be identified:

- During the early part of the first night, when the CAP was relatively thick and the upper‐level foehn flow was moderate, shear‐induced gravity waves formed in the CAP. They were visible as regular oscillations in the vertical velocity inside the CAP and associated alternating convergence and divergence in the horizontal near‐surface wind field. Later in the night, when the foehn had intensified and the wind shear between the CAP and foehn had increased, these oscillations were superimposed by smaller fluctuations suggesting shear‐induced turbulent mixing.
- A transient breakthrough occurred in the afternoon of 4 November. Increased turbulence and subcritical bulk Richardson number (${R\;i}_{b}$ < 0.25) suggest that shear flow instabilities contributed to this event. However, as the breakthrough occurred late in the afternoon and the CAP re‐established after sunset, despite the prevailing strength of the foehn, shear‐induced mixing at the top of the CAP was a secondary process and bottom‐up heating by the surface sensible heat flux a primary process in the destruction of the CAP on the first day.
- In the second night the CAP was shallower and the evolution of a breaking KH wave could be directly observed with coplanar scans of two Doppler wind lidars. The vertical amplitude of the breaking wave and the radius of a subsequent symmetric vortex were both about 300 m. However, it is likely that the full extent of this breaking wave could not be captured due to the limited vertical range of the lidar.
- The breakthrough on the last day occurred first in the northeastern part of the city before sunrise. Hence, the effect of solar radiation on CAP destruction was negligible. The CAP was shallow and therefore prone to displacement by wind stress. Consequently, the primary process of breakthrough at this stage was CAP displacement by a foehn branch which was deflected eastward at the Nordkette and finally penetrated into the CAP from the northeast.
- After the final breakthrough, a rotor formed above the city. Reversed (northerly) flow was observed in the city centre up to a height of about 700 m above the valley floor. Subrotors were visible in the two‐dimensional wind field on a vertical transect across the Inn Valley.

The different processes were analysed in order to evaluate their contribution to the CAP's removal and regeneration. The main findings are:

- Flow deflection at the mountain range north of Innsbruck was found to be an important factor for the penetration of foehn into the Inn Valley. For both transient breakthrough periods, foehn air approached the valley bottom from the mountain range north of the city. At both times, this process was accompanied by updraughts in the foehn flow above the city.
- The westward progression of the foehn–CAP boundary across the city during the final breakthrough was not the result of a direct westward CAP displacement. The latter would be associated with easterly foehn winds pushing the CAP westward, which was not observed. Instead, weak northerly foehn winds were recorded after breakthrough. However, foehn displacement could be observed when the CAP pushed back and lifted the foehn air from the valley floor prior to the cold front passage.
- The role of shear flow instabilities in eroding the CAP was quantified for both nights by estimating the vertical turbulent heat flux as a function of height above the Inn Valley based on a parametrization after Deardorff (1980). During both nights, the vertical turbulent heat flux convergence/divergence led to warming in the CAP and cooling above. This turbulent heating/cooling was partly compensated by other mechanisms. In the the first night, the compensation was not sufficient to prevent the CAP from heating. In contrast, during the second night, the CAP cooled despite the presence of turbulent mixing. As a result of strong CAP heterogeneity in the second night, estimated temperature advection terms were exceptionally high. This suggests that temperature advection played a large role in the CAP's heat budget.

Our carefully designed sampling strategy illustrated the fine‐scale structure of the foehn breakthrough in the Inn Valley. It became clear that, in this complex orographic environment, foehn–CAP interaction processes are highly three‐dimensional and require comprehensive measurement strategies including lidar remote sensing, ideally using multiple lidars. Nevertheless, a complete quantitative three‐dimensional heat budget analysis is very difficult with current observing systems and with manageable effort and expense. Therefore, real‐case large‐eddy simulations must be the logical next step to quantify such processes and to clarify their case‐dependent contribution to the total CAP heat budget. To evaluate these simulations in this extremely complex and challenging environment, the dataset collected during the PIANO field campaign will be very valuable.

## APPENDIX 1

### Doppler lidar methods

#### Calculation of wind components from coplanar scans

In theory, Equations (2) and (3) can be solved when at least two radial velocity measurements are available for the same point in space and time but with different angles (e.g., different az for Equation (2)). Hence, at least two lidars have to measure from different directions simultaneously at the same location. Both requirements (simultaneous and collocated measurements) are not perfectly fulfilled with our scanning strategy. The maximum temporal offset between two measurements for an arbitrary point on the plane is given by the duration of a single scan, which is 30 s in our case. For this period we assume stationarity in order to derive $v_{h}$ and $v_{v}$. The problem of non‐collocated measurement points is solved by using a Cartesian grid with a lattice length Δl=30 m which spans over the scan plane. For each grid point, the $v_{r}$ measurements which lie within the radius of influence $R=\Delta l/\sqrt{2}$ are collected. Using this definition for the radius of influence, measurement points are possibly assigned to more than one grid point. However, it is ensured that all measurements on the scan plane are used (Stawiarski *et al*., 2013). For the scanning strategies performed during the PIANO campaign (Table 1), on average four radial velocity measurements per lidar fall into one grid cell (for both vertical and horizontal coplanar scans). If N is the number of measurements within R, a system of N linear equations for each grid point is attained. This system can be solved by minimizing its cost function $J(v_{p})$ (Stawiarski *et al*., 2013). Since this approach is equal for the vertical and horizontal plane, $v_{p}$ is used here as a placeholder for $v_{h}$ and $v_{v}$. The associated unit vector is labelled as ${\hat{r}}_{p}$. Thus, the cost function takes the following form (Stawiarski *et al*., 2013):

(A1) $$J(v_{r})=\overset{N}{\underset{n=1}{\sum }}W_{n}{\left(v_{r,n}-{\hat{r}}_{p,n}\cdot v_{p}\right)}^{2}.$$

Here, $W_{n}$ are individual relative weights. One possible way of choosing $W_{n}$ is to consider the distance between individual measurements to the grid point, the so‐called inverse distance weighting (e.g., van Dooren *et al*., 2016). We tried this method, but for our dataset the difference between inverse distance and no distance weighting was negligible. However, in this work we chose $W_{n}$ to be inversely proportional to the number of measurements from a certain lidar. Firstly, the sum over all relative weights $W_{n}$ of all measurements within the radius of influence is always one. Secondly, the weights are equally distributed to the number of lidars (in our case two or three) that contribute to a single velocity estimate. Thirdly, these weights are equally distributed to the individual measurements of a certain lidar. This ensures that each lidar gets the same weight, regardless of the number of measurements it contributes to a single velocity estimate. The Python code of this algorithm is hosted at GitHub (https://github.com/marenha/doppler_wind_lidar_toolbox; accessed 8 January 2020). The GitHub repository is coupled to Zenodo, which provides DOIs for released software. The version of the code used in this study is version 1.0.0 (Haid, 2019).

#### Random error of Doppler lidar radial velocity

The random error of the velocity estimator, ϵ, is visible as white noise in the spectra of the radial velocity, $v_{r}$, and leads to an overestimation of its variance. The magnitude of ϵ depends on the corresponding signal‐to‐noise ratio (SNR) which is provided for each radial velocity estimate by the algorithm performed by the Stream Line software. With these SNR values, ϵ can be derived using a statistical model proposed by Rye and Hardesty (1993) and adjusted to Stream Line lidars by Pearson *et al*. (2009):

(A2) $$\epsilon =4\frac{\sqrt{\pi }}{\alpha }{\left(1+0.16\alpha \right)}^{2}\left(\frac{\Delta \nu^{2}}{N_{p}}\right).$$

The accumulated photocount, $N_{p}$, can be determined with

(A3) $$N_{p}=Mn\text{SNR},$$

and α is a dimensionless parameter that characterizes the ratio of the photon count to the speckle count. It is given by

(A4) $$\alpha =\frac{\text{SNR}}{\sqrt{2\pi \frac{\Delta \nu }{B}}}.$$

Here, n is the number of accumulated pulses, M the number of photon counts determining the range gate length, SNR represents the signal‐to‐noise ratio, B the bandwidth, and Δν the signal spectral width. Most of the parameters are predefined settings (M, n, B) or outputs of the data processing program (SNR). For a range gate length of 18 m, M=6 for the SL88, SL75 and SL74 lidars and M=12 for the SLXR142 lidar. A accumulation time of 1 s leads to n=15,000 (SL88, SL75 and SL74) and n=10,000 (SLXR142) (Section 2.1). The bandwidth, B, gives the range of resolvable radial velocities and dependents on the sampling frequency of the receiver. With sampling frequencies of 100 MHz (SLXR142) and 50 MHz (SL88, SL75 and SL74), B takes values of 75 and 37.5 m·s${}^{-1}$, respectively. Only for the signal spectral width, Δν, no unique definition was found. Therefore Δν was determined for each lidar using the measured $v_{r}$ and SNR values. This is done by estimating the ϵ values experimentally for each instrument and fitting Equation (A2) (SNR is independent variable) with Δν as free parameter. With the measured data, ϵ is estimated using the covariance method suggested by Frehlich (2001). The use of this method is most straightforward for time series of vertical stare data. The resulting values of Δν are than used in Equation (A2) which can be applied on every single measurement and therefore to other scan strategies like RHI and PPI. For the lidars used in this work, Δν is found to be 2.4 s${}^{-1}$ (SLXR142), 2.0 s${}^{-1}$ (SL75), 2.1 s${}^{-1}$ (SL74) and 2.0 s${}^{-1}$ (SL88).
